# Identification and validation of ferroptosis-related biomarkers in intervertebral disc degeneration

**DOI:** 10.3389/fcell.2024.1416345

**Published:** 2024-09-16

**Authors:** Chenglong Li, Chengshuo Fei, Shiyong Le, Zhongming Lai, Bo Yan, Liang Wang, Zhongmin Zhang

**Affiliations:** ^1^ Division of Spine Surgery, Department of Orthopedics, Nanfang Hospital, Southern Medical University, Guangzhou, China; ^2^ Department of Orthopedics, The Third Affiliated Hospital, Southern Medical University, Academy of Orthopedics, Guangzhou, China

**Keywords:** intervertebral disc degeneration, ferroptosis, bioinformatics analysis, immune cell infiltration, single-cell RNA sequencing

## Abstract

**Introduction:**

Ferroptosis plays a significant role in intervertebral disc degeneration (IDD). Understanding the key genes regulating ferroptosis in IDD could reveal fundamental mechanisms of the disease, potentially leading to new diagnostic and therapeutic targets.

**Methods:**

Public datasets (GSE23130 and GSE70362) and the FerrDb database were analyzed to identify ferroptosis-related genes (DE-FRGs) involved in IDD. Single-cell RNA sequencing data (GSE199866) was used to validate the specific roles and expression patterns of these genes. Immunohistochemistry and Western blot analyses were subsequently conducted in both clinical samples and mouse models to assess protein expression levels across different tissues.

**Results:**

The analysis identified seven DE-FRGs, including *MT1G*, *CA9*, *AKR1C1*, *AKR1C2*, *DUSP1*, *CIRBP*, and *KLHL24*, with their expression patterns confirmed by single-cell RNA sequencing. Immunohistochemistry and Western blot analysis further revealed that *MT1G*, *CA9*, *AKR1C1*, *AKR1C2*, *DUSP1*, and *KLHL24* exhibited differential expression during the progression of IDD. Additionally, the study highlighted the potential immune-modulatory functions of these genes within the IDD microenvironment.

**Discussion:**

Our study elucidates the critical role of ferroptosis in IDD and identifies specific genes, such as *MT1G* and *CA9*, as potential targets for diagnosis and therapy. These findings offer new insights into the molecular mechanisms underlying IDD and present promising avenues for future research and clinical applications.

## 1 Introduction

Intervertebral disc degeneration (IDD) is a leading cause of disability worldwide. It accounts for a significant proportion of lower back pain episodes globally (Andersson, 1999; Chen S. et al., 2021). A large portion of adults experience lower back pain at some point in their lives. Beyond diminishing the patient’s quality of life, this affliction exerts a profound economic strain on healthcare systems and society. IDD is driven by complex mechanisms including genetic predispositions, which indicate a hereditary vulnerability; mechanical stress from activities such as heavy lifting that strain the spine; cellular apoptosis, leading to a decline in the cells essential for disc maintenance; and obesity, which not only increases the load on the discs but also exacerbates degeneration through inflammatory and biochemical pathways. Each factor contributes uniquely to the progression of IDD. Contemporary therapeutic strategies, be they conservative or surgical, predominantly focus on palliating the associated pain but fall short in halting or reversing the degenerative process of IDD (Ma et al., 2022; Xin et al., 2022). Consequently, there’s an imperative to channel research toward identifying therapeutic strategies at the molecular or cellular level for IDD. This could herald a paradigm shift in treatment modalities, not only preserving the intrinsic biological functionality of the lumbar intervertebral discs but also significantly curtailing the prevalence of lower back pain.

In recent years, ferroptosis, a distinct form of cell death linked to oxidative stress and dysregulation of iron metabolism, has received significant attention. Its involvement is recognized in numerous diseases, particularly neurodegenerative disorders and cancers (Wei et al., 2022; Masaldan et al., 2019; Ge et al., 2022). There has been marked progress in understanding the role of ferroptosis in degenerative diseases, with many such diseases confirmed as related to ferroptosis (Yang et al., 2021). Considering that IDD is a degenerative condition marked by pronounced cell death, researchers are examining the potential importance of ferroptosis in IDD progression (Yu et al., 2022; Wang et al., 2022). However, current studies on ferroptosis in IDD are still nascent, necessitating further inquiry to clarify the associated pathways in intervertebral disc cells and prospective gene-targeted treatments. Ferroptosis’s interaction with the immune system is crucial for understanding IDD progression. Ferroptosis both influences and is influenced by the immune system, directly impacting the progression of IDD. For instance, macrophages play a central role in this interaction by releasing inflammatory cytokines such as TNF-α and IL-1β, which not only drive inflammation but also enhance ferroptosis, leading to increased matrix breakdown and exacerbating degeneration. Additionally, ferroptosis can activate immune regulation, where immune cells release cytotoxic substances and apoptotic signals, further promoting disc cell death and accelerating the progression of IDD. This dynamic interplay between ferroptosis and the immune system creates a feedback loop that worsens the degenerative process (Capossela et al., 2014; Ye et al., 2022; Zhang F. et al., 2023). Despite the significance of these interactions, research on immune cell infiltration during IDD progression remains limited, and the exact interplay between ferroptosis and immune infiltration in IDD has yet to be fully elucidated (Zhang et al., 2021; Wang et al., 2021).

Bioinformatics provides a powerful tool for uncovering complex disease mechanisms through the analysis of vast gene and protein databases. This approach is crucial for connecting the clinical features of IDD with their biological underpinnings. By using computational tools to study genetic and protein data, researchers can identify key biomarkers and targets for therapy, linking clinical outcomes with molecular discoveries to improve understanding and treatment options.

In our research, we used bioinformatics tools to conduct a comprehensive analysis that included Gene Ontology (GO) enrichment, KEGG pathway elucidation, and immune infiltration evaluation. The credibility of the identified DE-FRGs was rigorously tested through validation across varied datasets and Receiver Operating Characteristic (ROC) curve assessments. Subsequently, single-cell databases were employed to verify these genes and ascertain their expression in specific cell subtypes. Ultimately, the expression of these genes in clinical and mouse specimens was identified through immunohistochemistry and Western blotting, revealing key genes associated with ferroptosis in disc degeneration. These findings pave the way for novel therapeutic strategies targeting ferroptosis in IDD.

## 2 Materials and methods

### 2.1 Data collection and preprocessing

Following download and filtration processes, microarray data and platform annotation were sourced from the GEO datasets GSE23130 and GSE70362. GSE23130 was designated as the training dataset, while GSE70362 was used as an external dataset. Data analysis was conducted using the R programming language (version4.1.1).

### 2.2 Differential expression analysis of ferroptosis-related genes

The repeatability of the microarray data was assessed using principal component analysis. Differential analysis was executed using the “limma” package (Ritchie et al., 2015). For GSE23130, genes with |log2FC|>1 and *P*-value <0.05 were identified as differentially expressed genes (DEGs). In contrast, for GSE70362, the criteria were |Foldchange|>1.5 and *P*-value < 0.05. The “heatmap” and “ggplot2” packages (Steenwyk and Rokas, 2021) were utilized to generate heatmaps, volcano plots, and box plots. DE-FRGs were derived from the overlap between DEGs and ferroptosis-related genes (FRGs), with Venn plots illustrating the results.

### 2.3 Enrichment analyses

Enrichment analyses included Gene Ontology (GO) (The Gene Ontology, 2004) and Kyoto Encyclopedia of Genes and Genomes (KEGG) (Kanehisa and Goto, 2000) methodologies using the “clusterProfiler” package (Yu et al., 2012). GO enrichment delved into biological processes (BP), cellular components (CC), and molecular functions (MF), with significance defined by an adjusted P-value < 0.05. The “clusterProfiler” package also facilitated Gene Set Enrichment Analysis (GSEA) (Subramanian et al., 2005), while visualization was achieved with the “enrichplot” package. The C5 GO gene sets database (c5.all.v7.1.symbols.gmt) from the Molecular Signatures Database (MSigDB) (Liberzon et al., 2011) helped pinpoint significant biological processes between control and IDD groups. Similarly, the C2 curated gene sets (c2.all.v7.1.symbols.gmt) from MSigDB illuminated enriched signaling pathways. Significant functional enrichment was discerned with thresholds of FDR < 0.25 and *P* < 0.05.

### 2.4 Identification and verification of key genes

We validated the differential expression of candidate key DE-FRGs using an external dataset and identified the key DE-FRGs with specific expressions in AF and NP. In the receiver operating characteristic (ROC) analysis, we evaluated the diagnostic potential of these key DE-FRGs using the “pROC” package. Ideally, the Area Under the Curve (AUC) should fall between 0.5 and 1; an AUC approaching 1 indicates superior diagnostic quality.

### 2.5 Single-cell RNA-Seq data processing

The GSE199866 dataset was downloaded from the GEO database, comprising four samples including non-degenerated and degenerated NP and AF cells (NPnD, NPD, AFnD, AFD), totaling 14,001 cells. Data preprocessing and subsequent analyses were performed using the Seurat (v4.3.0) R package. Quality control parameters were set as follows: the number of features greater than 300 or less than 8,000; cells with count numbers between 500 and 60,000 were retained; cells with mitochondrial gene expression percentages over 20% and a hemoglobin proportion higher than 10% were removed. Following this, data normalization was conducted, principal component analysis (PCA) was applied for dimensionality reduction, and batch effects were mitigated using Harmony. Clustering and visualization were performed using the UMAP method with a resolution of 0.5. Clusters were identified using Seurat’s Find All Clusters function. Gene Set Variation Analysis (GSVA) was utilized to analyze pathway activities in clusters from single-cell transcriptomes, utilizing “c5.go.v2023.2.Hs.symbols.gmt” gene sets from MSigDB. Pseudotime analysis and visualization were performed using Monocle2.

### 2.6 Clinical data acquisition and specimen collection

Following approval from our hospital’s ethics committee, 24 discarded intervertebral disc tissue samples were collected from patients undergoing posterior lumbar discectomy at the Department of Spine Surgery. All participating patients were informed about the study and provided written consent.

All tissue samples were sourced from either the L4/5 or L5/S1 segments and were subjected to magnetic resonance imaging (MRI) assessments. The degree of disc degeneration was evaluated using the Pfirrmann score (Pfirrmann et al., 2001) based on pre-surgery MRI findings. In this grading system, grade I signifies no degeneration, grades 2–3 denote mild degeneration, and grades 4–5 indicate severe degeneration. For the purposes of this study, samples with grades 2–3 were categorized into the mild-IDD group, while those with grades 4–5 were grouped into the severe-IDD category.

### 2.7 Intervertebral disc degeneration mouse model

This study involved twenty-four 8-week-old C57 mice, used under the approval of our institution’s animal experimentation committee. The mice were randomly assigned into two groups: a control group (Control) and an experimental group (IDD). The experimental protocol involved placing each mouse in a specialized device within a large cage, partially filled with 5 mm of water. This environment encouraged the mice to maintain a bipedal stance for 6 h each day over a period of 1 week. Outside of these hours, the mice were allowed free movement and access to food and water. This regimen was designed to exert significant stress on the lumbar spine, thereby inducing lumbar IDD (Ao et al., 2019). The experiment lasted for 12 weeks, after which all mice were humanely euthanized for further analysis.

### 2.8 Histological and immunohistochemistry studies

In the histological analysis phase, each tissue sample was initially fixed in 4% paraformaldehyde, then decalcified, embedded in paraffin, and finally sectioned into 4-μm thick slices. These prepared sections underwent deparaffinization and rehydration, followed by staining with hematoxylin and eosin (H&E), Masson’s trichrome, and safranin O/Fast green (SOFG) in accordance with the manufacturer’s guidelines. For the histological grading, we used the Weiler et al. (2011) scale for clinical specimens and the Tam et al. (2018) scale for mouse specimens, with three independent researchers performing the grading for each group.

The immunohistochemistry (IHC) analysis involved several key steps. After the deparaffinization and rehydration of paraffin sections, antigen retrieval was conducted by microwaving human tissue sections in EDTA buffer (pH 8) and mouse sections in citrate buffer (pH 6) for 3 min. We quenched endogenous peroxidase activity by treating the sections with 3% hydrogen peroxide for 15 min in a dark environment. To block non-specific binding, the sections were incubated with goat serum (AR0009, Boster, China) for 1 h. Following this, sections were incubated overnight at 4°C with the primary antibodies, specifically GPX4 (1:100; T56959, Abmart), MT1G (1:100; LS-B13009, BioSciences), CA9 (1:100; T55592, Abmart), DUSP1 (1:100; T56588, Abmart), AKR1C1/2 (1:100; T58076, Abmart), and KLHL24 (1:100; PU160205, Abmart). Subsequently, they were treated with goat anti-rabbit IgG (H + L) HRP secondary antibody (BF03008X, Bio-dragon) for 2 h at room temperature. Visualization was achieved using DAB (Service-Bio, Shanghai, China), and hematoxylin was used for counterstaining. The stained sections were examined under an Olympus BX63 microscope (Olympus, Tokyo, Japan), and the proportion of positive staining was quantitatively assessed using Image J software (NIH, United States).

### 2.9 Western blot

Clinical IVD specimens (mild-idd group: severe-idd group = 2:2), alongside mouse IVD specimens (control group: idd group = 2:2), were central to this study. All samples were immediately frozen in liquid nitrogen and stored at −80°C before Western blot analysis. Tissues were lysed with RIPA buffer, and proteins were resolved via SDS-PAGE, transferred to PVDF membranes, and probed with primary antibodies, including GPX4, *MT1G*, *CA9*, and GAPDH. Detection was performed using chemiluminescence, and signal quantification was facilitated by Image Lab software.

### 2.10 Immune cell infiltration estimation

CIBERSORT is a specialized deconvolution method that discerns and quantifies the infiltration of 22 distinct immune cell subtypes (Newman et al., 2015). Using the CIBERSORTR script, we assessed the abundance of these 22 immune cells in the GSE23130 dataset. Visualization of results was facilitated through the “vioplot” package. To determine the correlations between infiltration rates of different immune cell types, we used the “complot” package. Spearman’s correlation analysis was executed on pivotal DE-FRGs and infiltrating immune cells employing the “ggpubr” and “ggExtra” packages. Further, we utilized the Wilcoxon signed-rank test to identify the differentially infiltrated immune cells (DIICs) in the IDD group in contrast to the control group, with significance set at *P* < 0.05.

### 2.11 Statistical analysis

Statistical evaluations in this study were conducted using various software tools: R (version 4.1.0), SPSS 20.0 (SPSS Inc., Chicago, IL, United States), and GraphPad Prism 9.0.0 (GraphPad Software, La Jolla, CA, United States). Data representation was in the format of mean ± standard error of the mean for all the parameters measured. For statistical comparisons, Student’s t-tests were applied, considering *P* < 0.05 as the threshold for statistical significance.

## 3 Results

### 3.1 Screening of DEGs and identification of DE-FRGs

**FIGURE 1:**
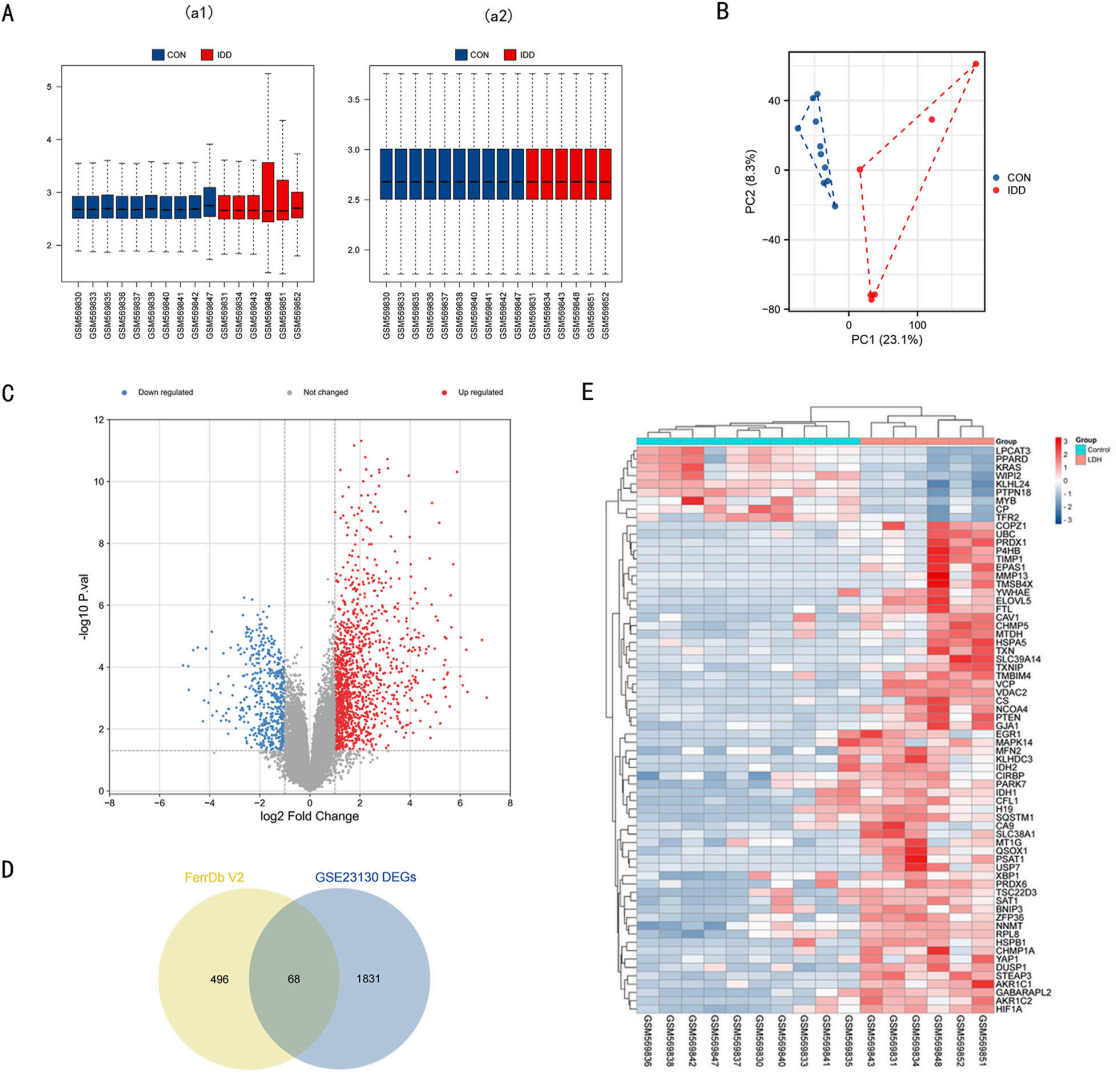
Data preprocessing of microarray data and DE-FRGs in GSE23130. **(A)** a1, The boxplot of GSE23130 before sample data standardization. a2, The boxplot of GSE23130 after sample data standardization. **(B)** Principal component analysis of the microarray data set GSE23130. **(C)** Volcano plot of GSE23130. Red refers to upregulated expression. Blue refers to downregulated ex-pression. Gray indicates no difference in expression. **(D)** Venn diagrams indicating 68 DE-FRGs. Blue indicates the 1899 DEGs. Yellow indicates the 564 FRGs. **(E)** Heatmap of the 68 DE-FRGs in Intervertebral disc.

In this section, we describe the process of identifying differentially expressed genes (DEGs) and ferroptosis-related genes (DE-FRGs) involved in intervertebral disc degeneration (IDD). Microarray data GSE23130 was sourced from the GEO database. After normalization (Figure 1A), we conducted PCA (Principal Component Analysis) as depicted in Figure 1B. Based on the PCA outcomes, we eliminated outlier samples and subsequently considered ten normal and 6 IDD samples from the dataset for further examination. The differential expression analysis revealed 1899 DEGs: 544 downregulated and 1,355 upregulated, visualized in the volcano plot (Figure 1C). Using the FerrDb V2 database, we retrieved a set of 564 FRGs. Their intersection with the 1899 DEGs yielded 68 ferroptosis-related DEGs, depicted in the heatmap (Figures 1D, E). Detailed data regarding these 68 DE-FRGs can be found in Supplementary Table 1.

### 3.2 Enrichment analyses of DE-FRGs

**FIGURE 2:**
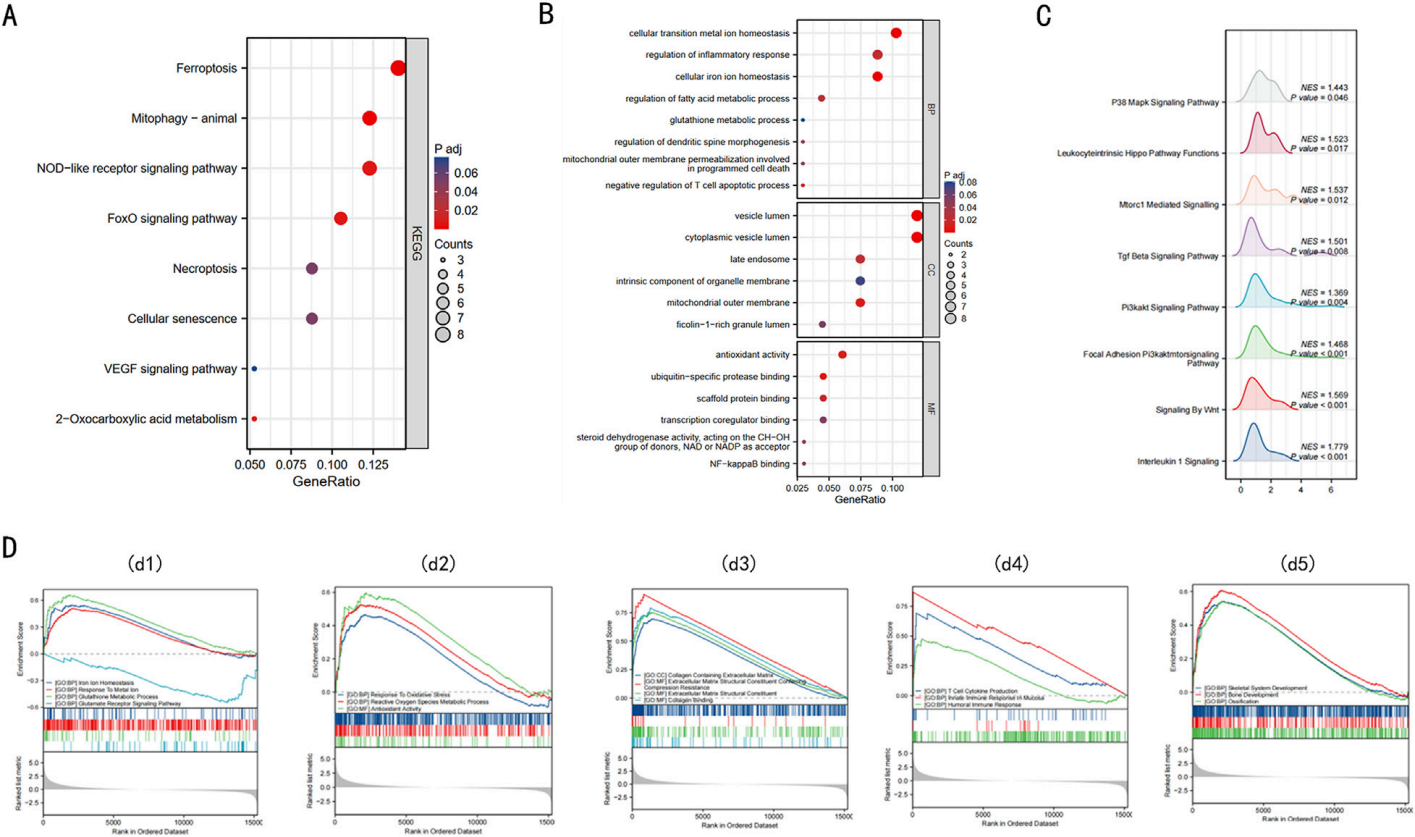
Enrichment analysis of 68 DE-FRGs in Intervertebral disc. **(A)** The bubble plot displays the gene Ontology (GO) enrichment analysis of 68 DE-FRGs in the Intervertebral disc. **(B)** The bubble plot shows the Kyoto Encyclopedia of Genes and Genomes (KEGG) analysis of 68 DE-FRGs in the Intervertebral disc. **(C)** Ridge plot of GSEA results for C2 pathway. **(D)** d1, The GSEA enrichment plot indicates that BPs relating to ferroptosis are enriched in the IDD group. d2, The GSEA enrichment plot indicates that BPs relating to ROS are enriched in the IDD group. d3, The GSEA enrichment plot indicates that BPs relating to collagen are enriched in the IDD group. d4, The GSEA enrichment plot indicates that BPs relating to immunity are enriched in the IDD group. d5, The GSEA enrichment plot indicates that BPs relating to bone development are enriched in the IDD group.

To elucidate the biological functions of the 68 DE-FRGs, we undertook KEGG and GO analyses. The KEGG analysis spotlighted significant engagement of pathways like Ferroptosis, NOD-like receptor, FOXO, and VEGF (Figure 2A) (refer to Supplementary Table 2).

The GO enrichment analysis provided insights into the functions of differentially expressed ferroptosis-related genes (DE-FRGs), highlighting their involvement in key processes that are linked to iron-induced cell death or ferroptosis. In the Biological Process (BP) category, these genes were primarily involved in maintaining cellular iron ion homeostasis, glutathione metabolism, and regulating fatty acid metabolism, all of which are crucial for controlling the iron levels and oxidative state within cells. Furthermore, they were involved in mitochondrial outer membrane permeabilization, a critical event in programmed cell death that can be triggered by iron overload and lead to cell death. In the Cellular Component (CC) category, there was notable enrichment in structures like the mitochondrial outer membrane and the late endosome, which are essential in managing cellular iron distribution and could play roles in initiating ferroptosis if dysregulated. Lastly, in the Molecular Function (MF) category, associations with antioxidant activity, NF-κB binding, and ubiquitin-specific protease binding suggest mechanisms by which these genes might influence ferroptosis. Antioxidant activity is crucial in mitigating oxidative stress caused by iron, while NF-κB and ubiquitin-specific proteases could regulate the cellular response to stress and damage, including ferroptosis (Figure 2B) (details in Supplementary Table 3). These findings collectively underscore the complex interplay of molecular functions that govern iron-induced cell death, linking cellular iron management with the pathways leading to ferroptosis.

GSEA, utilizing C2 gene sets, was illustrated via a ridge plot (Figure 2C), highlighting pathways like INTERLEUKIN 1 SIGNALING, SIGNALING BY WNT, PI3KAKT SIG-NAL-ING PATHWAY, these pathways are known to be involved in inflammation, cell proliferation, and survival, which can be crucial in the context of IDD progression. Subsequent GSEA enrichment plots for GO gene sets confirmed the predominance of processes like iron ion homeostasis, response to metal ions, and glutathione metabolism in the IDD group (Figure 2d1). These processes are directly linked to managing iron levels and detoxifying reactive oxygen species (ROS), implicating ferroptosis could significantly contribute to IDD pathology. Additionally, there were discernible enrichments related to ROS (Figure 2d2), collagen (Figure 2d3), immunity (Figure 2d4), and bone development (Figure 2d5) within the IDD group. A comprehensive summary of the GSEA findings is provided in Supplementary Table 5. Obtain seven key DE-FRGs in Nucleus Pulposus (NP) and Annulus Fibrosus (AF), these genes are essential for understanding the molecular mechanisms of IDD and how ferroptosis might be specifically managed in these tissues to potentially mitigate disease progression.

**FIGURE 3:**
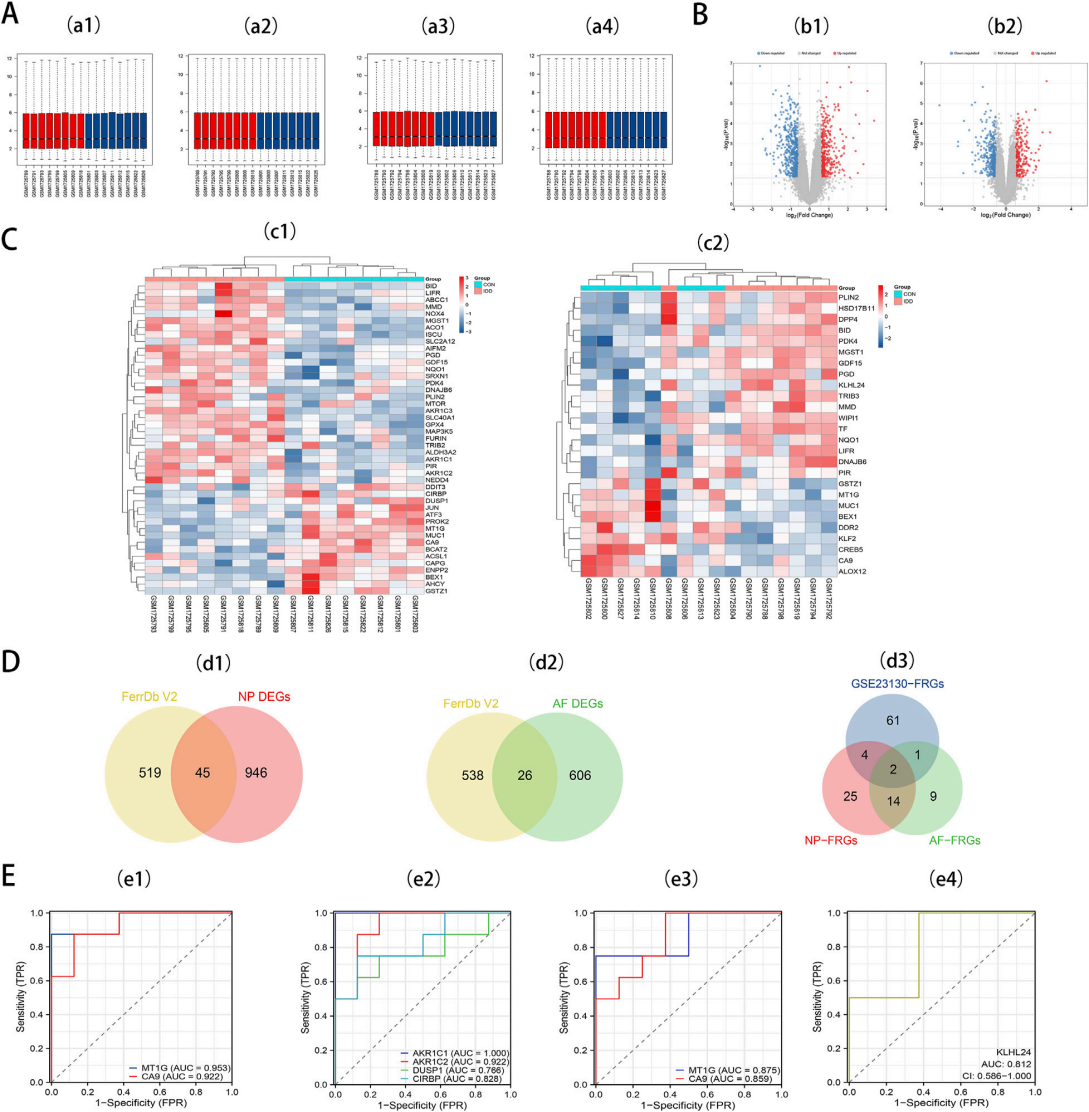
**(A)** a1, The boxplot of GSE70362 before AF sample data standardization. a2, The boxplot of GSE70362 after AF sample data standardization. a3, The boxplot of GSE70362 before NP sample data standardization. a4, The boxplot of GSE70362 after NP sample data standardization. **(B)** b1, Volcano plot of DEGs in AF. b2, Volcano plot of DEGs in NP. Red refers to upregulated expression. Blue refers to downregulated expression. Gray indicates no difference in expression. **(C)** c1, Heatmap of the 45 DE-FRGs for NP. c2, Heatmap of the 26 DE-FRGs for AF. **(D)** Venn diagrams indicate 45 DE-FRGs for NP and 26 DE-FRGs for AF. Blue indicates the 68 DE-FRGs derived from the Intervertebral disc dataset. Yellow indicates the 564 FRGs, Red indicates 45 DE-FRGs for NP, and Green indicates 26 DE-FRGs for AF. **(E)** e1, ROC curves of *MT1G*, and *CA9* in NP tissues. e2, ROC curves of NP-specific DE-FRGs. e3, ROC curves of *MT1G*, and *CA9* in AF tissues. e4, ROC curves of AF-specific DE-FRGs.

We retrieved the GSE70362 microarray expression profiling dataset from the GEO database, utilizing it as a validation set. From this, 16 fibrillar and 16 myeloid samples were chosen for normalization (Figures 3a1, 3a2). Subsequent differential expression analyses pinpointed 632 DEGs in AF and 991 DEGs in NP. For the DEGs in AF, 373 genes exhibited downregulation, while 259 showed upregulation. In contrast, the NP samples had 587 downregulated genes and 404 upregulated ones. These distributions are depicted in volcano plots (Figures 3b1, 3b2). By intersecting 564 FRGs with the identified DEGs in AF (632) and NP (991), we ascertained 26 DE-FRGs for AF (Figure 3d1) and 45 DE-FRGs for NP (Figure 3d2). The heatmaps showcasing these intersections can be observed (Figures 3c1, 3c2). More granular details on DE-FRGs in AF and NP are cataloged in Supplementary Table 5 and Supplementary Table 6, respectively.

To pinpoint diagnostic biomarkers integral to IDD, we carried out an intersection analysis on the 68 DE-FRGs derived from the Intervertebral disc dataset. This scrutiny illuminated two central DE-FRGs - *MT1G* and *CA9* - consistently expressed in both AF and NP regions. Intriguingly, *KLHL24* surfaced as a unique DE-FRG confined to AF, whereas NP revealed a distinct preference for four DE-FRGs: *AKR1C1*, *AKR1C2*, *DUSP1*, and *CIRBP* (Figure 3d3).

Seven key DE-FRGs was assessed via receiver operating characteristics (ROC) using an external dataset. In NP tissues, *MT1G* and *CA9* showcased AUC values of 0.953 and 0.922 (Figure 3e1). The ROC curve for the NP-specific DE-FRGs—*AKR1C1*, *AKR1C2*, *DUSP1*, and *CIRBP*—exhibited AUC values of 1.000, 0.922, 0.766, and 0.828, respectively (Figure 3e2). Meanwhile, in AF tissues, *MT1G* and *CA9* showcased AUC values of 0.875 and 0.859, respectively (Figure 3e3). The AF-specific DE-FRG, *KLHL24*, displayed an AUC value of 0.812 (Figure 3e4).

These enrichment analyses reveal the complex interplay of molecular functions and pathways that govern ferroptosis in IDD, offering potential targets for therapeutic intervention.

### 3.3 Confirmation of expression patterns of ferroptosis key genes in a single-cell dataset

To further explore the expression of key DE-FRGs at the single-cell level, we analyzed a single-cell RNA-seq dataset to identify the cell types and clusters expressing these genes in non-degenerated and degenerated intervertebral disc tissues.

**FIGURE 4:**
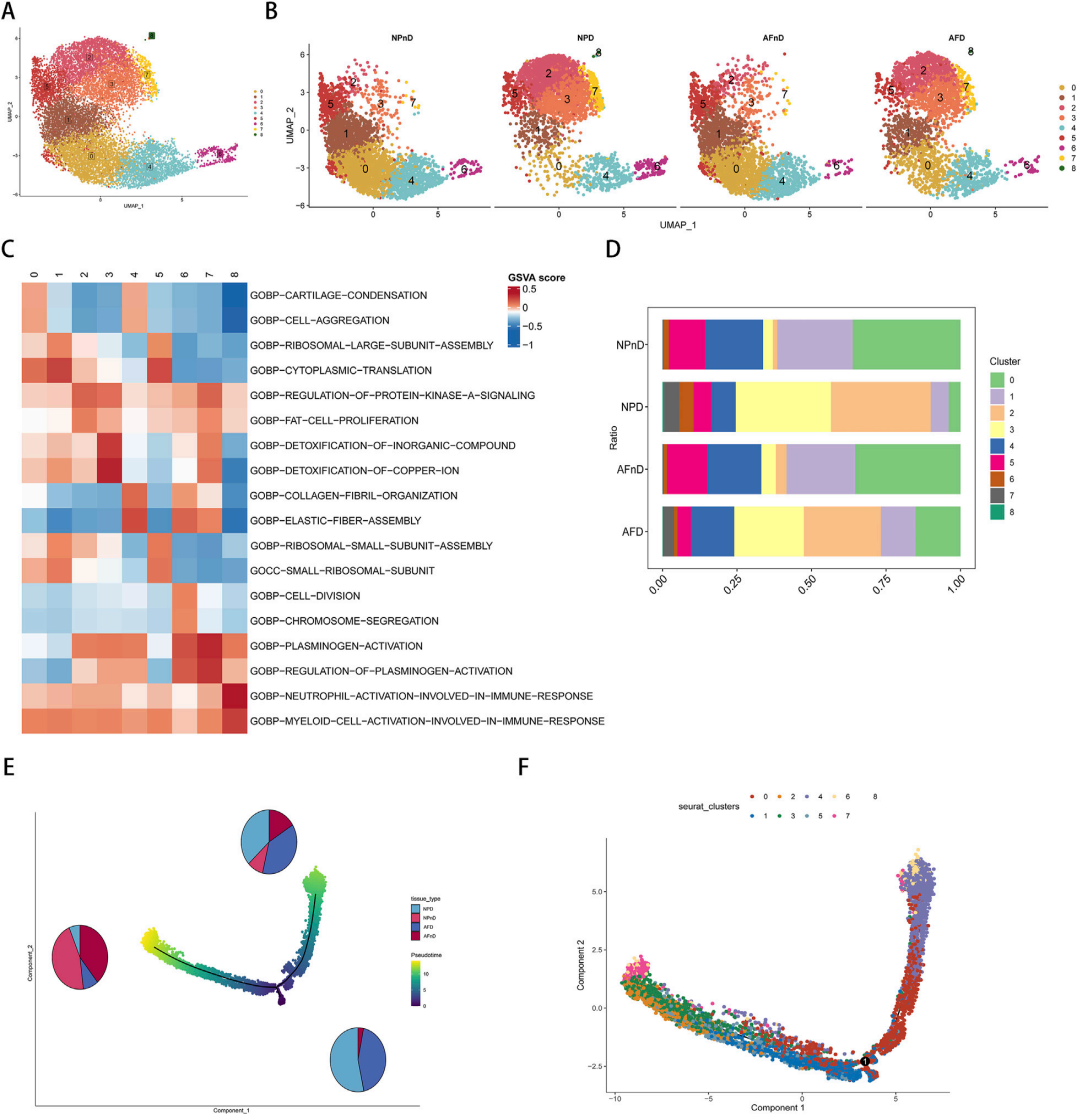
Single-cell transcriptomic landscape and pseudotime trajectory analysis of intervertebral disc cells. **(A)** Unsupervised UMAP projection representing the global transcriptional profiles of disc cells. **(B)** Unsupervised UMAP clustering showing the change in cell distribution of the 14 different clusters for NPnD, NPD, AFnD, and AFD. **(C)** GSVA enrichment analysis of the identified nine cell clusters, displaying the most representative metabolic pathways using a heatmap. **(D)** Bar chart showing the proportional representation of each cluster within the four conditions. **(E)** The pseudotime trajectory axis for tissue types along the progression of IVD degeneration, with pie charts. **(F)** The pseudotime trajectory axis for defined nine cell clusters.

Our analysis incorporated the GSE199866 single-cell RNA-seq dataset, which included 3,955 NP cells from non-degenerated discs (NPnD), 3,678 NP cells from degenerated discs (NPD), 3,226 AF cells from non-degenerated discs (AFnD), and 3,142 AF cells from degenerated discs (AFD). We analyzed the single-cell data after quality control and visualized all cells through UMAP plots (Figures 4A, B), revealing 9 clusters with distinct expression profiles, designated as clusters 0–8. This reflects the heterogeneity of intervertebral disc cell functions. These plots reveal the distinct transcriptional identities of cells in non-degenerated versus degenerated conditions, highlighting potential molecular pathways impacted by disc degeneration.

To identify the functions of each cluster and explain the role of DE-FRGs in IDD.GSVA enrichment analysis was performed on the single-cell transcriptomes, displaying the most representative metabolic pathways using a heatmap (Figure 4C). Additional results are presented in the Supplementary Table 7. Cluster 0 emphasizes the importance of cartilage and bone formation as well as the integrity of the extracellular matrix, which are vital for maintaining the structure and function of the intervertebral disc. The condensation of cartilage and the completeness of the extracellular matrix are crucial for resisting structural damage and functional loss during disc degeneration. Cluster 1 reveals the significance of protein synthesis, particularly the role of ribosomes, which is essential for repairing the extracellular matrix and combating degenerative stress in disc cells. Clusters 2 and 3, related to lipid metabolism and stress response, may be linked to the nutritional supply and cellular response mechanisms of the disc. Cluster 4 focus on cellular growth and differentiation, especially in bone and cartilage development, is significant for regenerative medicine and stem cell therapy strategies in disc research. Both Clusters 5 and 1 underscore the role of protein synthesis in restoring disc cell function and maintaining matrix integrity. Cluster 6 unveils the importance of cell division and chromosomal dynamics, potentially related to disc cell proliferation and genetic stability, which are essential for disc health and preventing degeneration. Cluster 7, involving blood coagulation and fibrinolysis, could offer insights into the inflammatory response and angiogenesis during disc degeneration. Lastly, Cluster 8 highlights the activation and regulation of specific immune cell types, unveiling potential immune-mediated mechanisms in disc degeneration, particularly in the study of disc inflammation and pain mechanisms.

In the intervertebral disc tissues, we observed an increase in the proportions of clusters 2, 3, and 7, and a decrease in clusters 0, 1, 4, and 5, potentially revealing critical biological changes and molecular mechanisms in the process of disc degeneration. The rising clusters indicate intensified inflammatory responses, heightened stress adaptation, and alterations in local microcirculation, while the declining clusters suggest a weakening in extracellular matrix synthesis and maintenance, protein synthesis, and the capacity for tissue repair and regeneration (Figure 4D).

The pseudotime analysis suggests a trajectory of cellular evolution within the degenerative process of intervertebral disc cells. The sequence or trajectory shown by cell clustering likely reflects the continuum of cell states, from healthy to degenerative stages. The proportions of cells along the principal component 1 axis (horizontal axis) align with the types of cells in disc degeneration, with clusters 0, 1, 4, and 5 representing the states of healthy or early degenerative disc cells, while clusters 2, 3, and 7 may correspond to more advanced stages of degeneration (Figures 4E, F).

**FIGURE 5:**
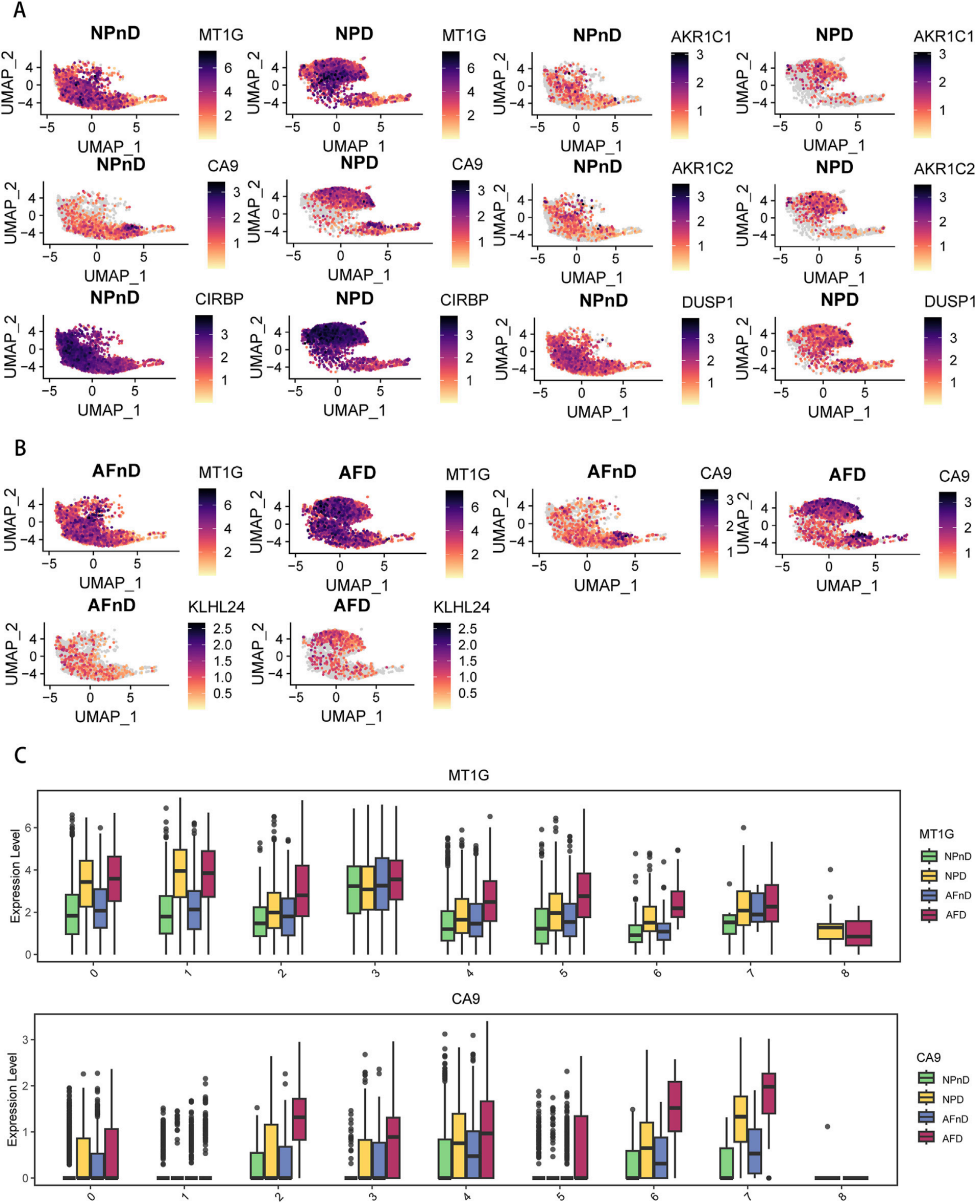
Expression of DE-FRGs in a single-cell dataset **(A)** UMAP plots demonstrate the expression levels of NP-specific DE-FRGs in NPnD versus NPD cells. **(B)** UMAP plots demonstrate the expression levels of AF-specific DE-FRGs AFnD versus AFD cells. **(C)** Box plots show the quantification of expression levels for *MT1G* and *CA9* across all clusters.

The results indicate a nuanced expression pattern of key genes across different states of intervertebral disc cells, as visualized in UMAP plots (Figures 5A, B). Notably, *MT1G* and *CA9* expressions vary across clusters in both NP and AF cells, with *MT1G* generally upregulated in degenerated tissues. This suggests an adaptive cellular response to degenerative stress, potentially involving metal ion balance and oxidative stress. *CA9* upregulation in specific clusters (clusters 2, 3, 6, and 7) aligns with responses to hypoxic and acidic environments, implicating its role in disc degeneration. Box plots further quantify these expression changes (Figure 5C). The expression pattern of GPX4 is shown in Supplementary Figure 1.

The single-cell analysis highlights the specific cellular environments where ferroptosis-related changes occur, underscoring the importance of these genes and pathways in the progression of IDD.

### 3.4 Validation of ferroptosis key genes in clinical specimens

**FIGURE 6:**
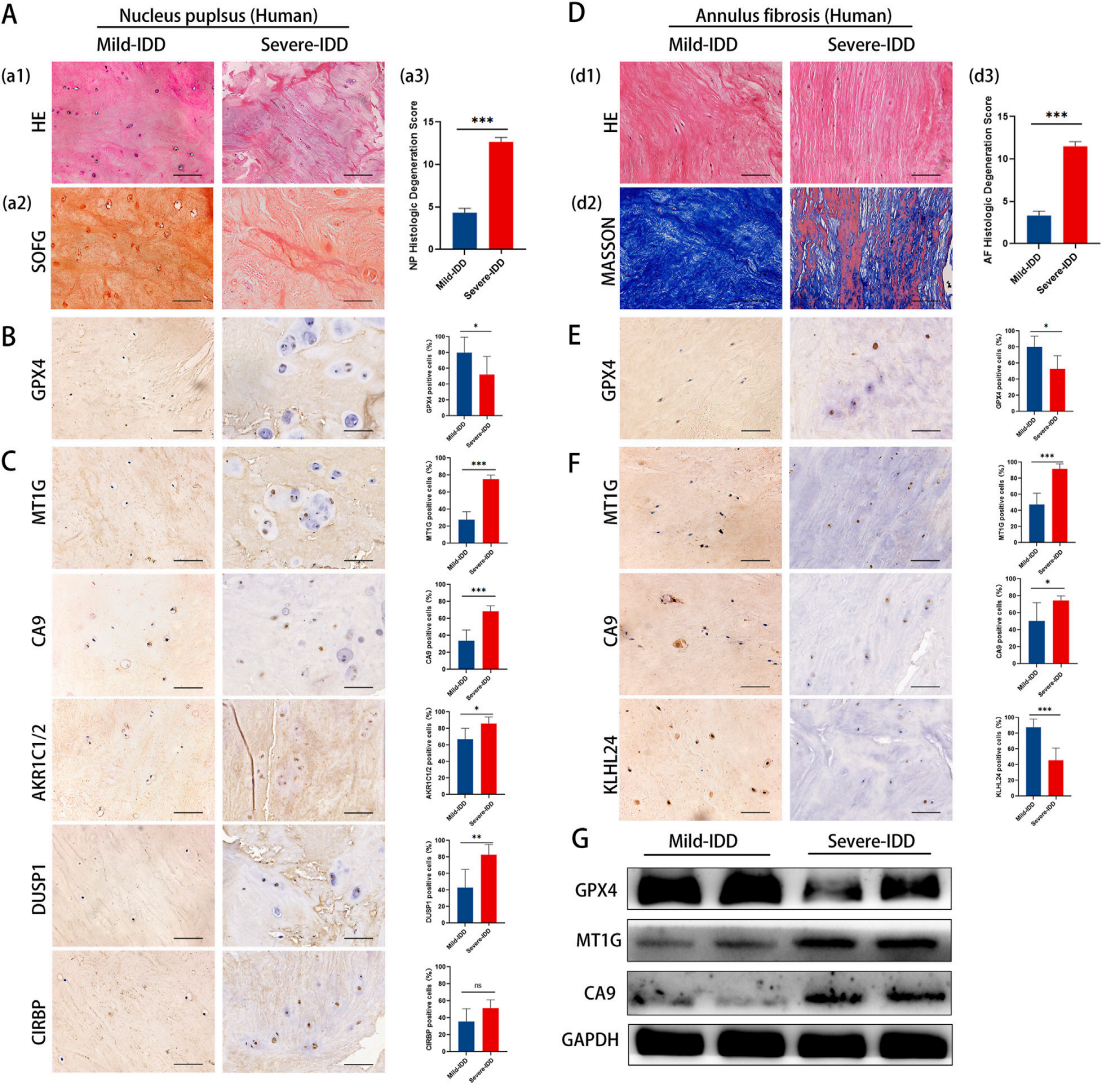
Representative histology and protein expression levels of clinical specimen. **(A)** HE staining, SOFG staining and histologic Degeneration Score of NP **(B)** Representative IHC images of GPX4 in NP. **(C)** Representative IHC images of *MT1G*, *CA9*, *AKR1C1*/2, *DUSP1*, and *CIRBP* in the NP. **(D)** HE staining, Masson staining and histologic Degeneration Score of AF. **(E)** Representative IHC images of GPX4 in AF. **(F)** Representative IHC images of *MT1GCA9*and *KLHL24* in the AF. **(G)** The protein levels of GPX4, *MT1G* and *CA9* of clinical specimen.

To validate our findings in a clinical context, we examined the expression of key DE-FRGs in clinical specimens categorized by the degree of disc degeneration.Clinical samples were categorized by Pfirrmann grading, detailed in Supplementary Table 8. Utilizing hematoxylin and eosin (HE) staining to examine cell density and structural alterations, the analysis revealed that the NP tissues in the mild-IDD group manifested a significantly higher density of chondrocyte cells. Conversely, the severe-IDD group was characterized by a substantial decrease in cell quantity and the emergence of small-sized cellular clusters (comprising 3-7 chondrocytes), accompanied by more pronounced structural disruptions. Additionally, Safranin-O/Fast Green (SOFG) staining uncovered a striking contrast in proteoglycan distribution, with the mild-IDD group displaying a consistent red matrix, markedly diminished in the severe-IDD group (Figure 6A). Further scrutiny of the annulus fibrosus (AF) elucidated a compromised structural integrity in discs with severe degeneration, manifesting as disorganized fiber patterns. This observation was corroborated by Masson’s trichrome staining, which further emphasized extensive red-stained regions, indicative of advanced degeneration (Figure 6D). The variation in histological degeneration scores between the groups significantly underscored the severe degenerative alterations observed in the severe group.

To clarify the correlation between ferroptosis and IDD, we examined GPX4 expression in clinical samples with different degrees of deformation in NP and AF. Immunohistochemistry results showed that the expression of *GPX4* was decreased in both NP and AF of the highly degenerated group, and the degree of ferroptosis increased with the degree of degeneration (Figures 6B, E).

The results of immunohistochemistry staining showed that in NP, the expression of *MT1G, CA9, AKR1C1/2, DUSP1* was significantly increased, whereas *CIRBP* expression remained unchanged (Figure 6C). In AF, the expression of *MT1G* and *CA9* increased significantly, while the expression of *KLHL24* decreased significantly (Figure 6F). Western blotting results showed that *MT1G* and *CA9* protein levels were upregulated in mild-IDD specimens compared with severe-IDD specimens, while the protein levels of *GPX4* were decreased (Figure 6G).

The results from clinical specimens confirm the differential expression of key ferroptosis-related genes, reinforcing their potential role in the pathology of IDD.

### 3.5 Validation of ferroptosis key genes in IDD mouse models

**FIGURE 7:**
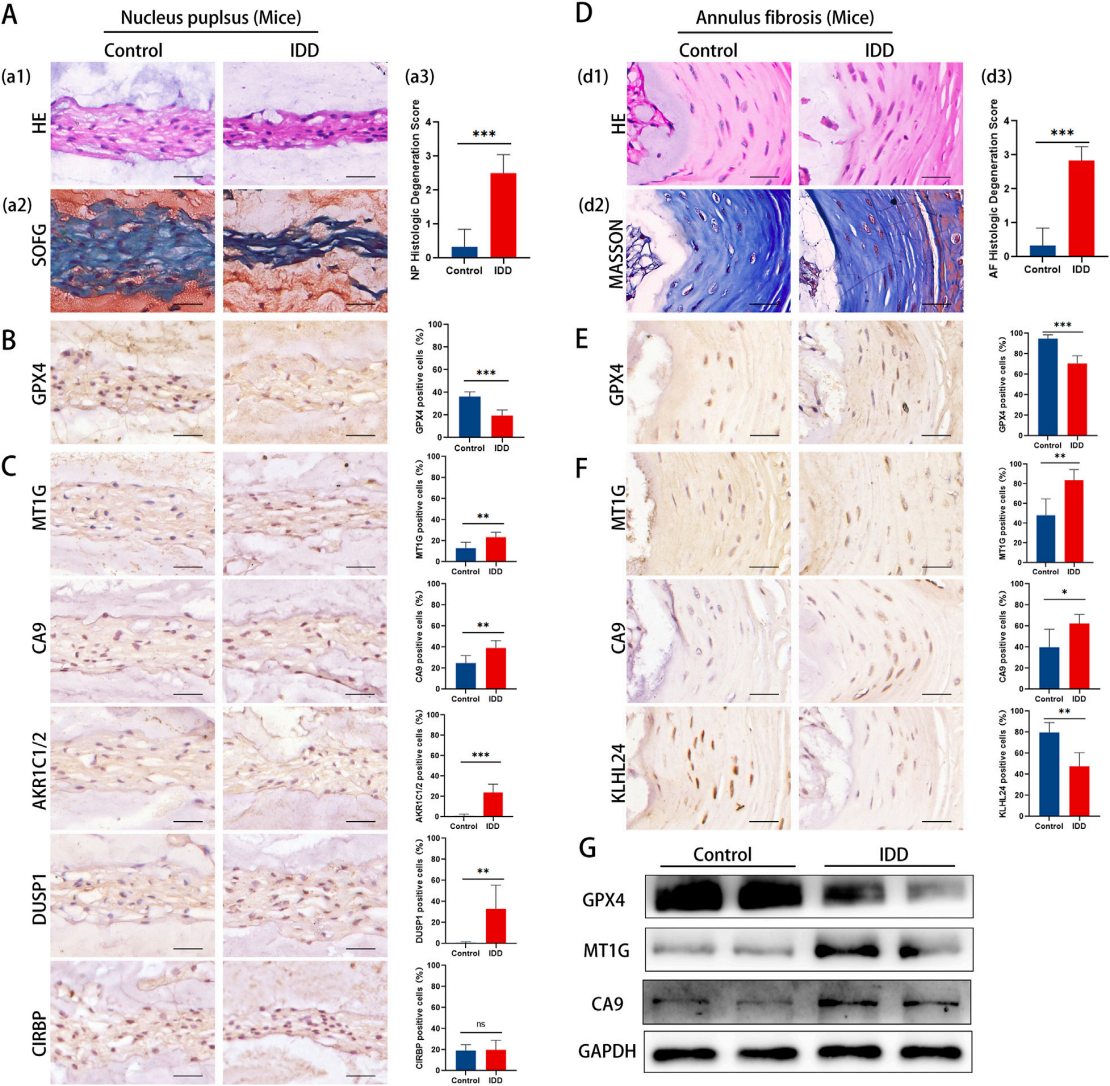
Representative histology and protein expression levels of mice specimen. **(A)** HE staining, SOFG staining and histologic Degeneration Score of NP **(B)** Representative IHC images of GPX4 in NP. **(C)** Representative IHC images of *MT1G*, *CA9*, *AKR1C1*/2, *DUSP1*, and *CIRBP* in the NP. **(D)** HE staining, Masson staining and histologic Degeneration Score of AF. **(E)** Representative IHC images of GPX4 in AF. **(F)** Representative IHC images of *MT1GCA9*and *KLHL24* in the AF. **(G)** The protein levels of GPX4, *MT1G* and *CA9* of mice specimen.

To further corroborate our findings, we investigated the expression of key DE-FRGs in IDD mouse models, focusing on histological and molecular changes.Histological evidence highlighted more severe degeneration in the IDD mouse group, showcasing a decrease in NP cells, extracellular matrix, and cell size (Figure 7A). Advanced degenerative changes were also noted in the annulus fibrosus (Figure 7D).

Immunohistochemistry analysis revealed a significant reduction in GPX4 expression in both NP and AF tissues (Figures 7B, E). The results aligned with clinical specimens.

The results of immunohistochemistry staining showed that in the NP, there was a notable upsurge in the expression of *MT1G, CA9, AKR1C1/2,* and *DUSP1*, whereas *CIRBP*’s expression remained largely unaltered (Figure 7C). Conversely, in the AF region, *MT1G* and *CA9*’s expression was markedly amplified, while *KLHL24*’s expression diminished significantly (Figure 7F). Western blotting confirmed these observations, showing a decrease in *GPX4* and an increase in *MT1G* and *CA9* in IDD mouse IVD tissues (Figure 7G).

The mouse model results align with our clinical findings, further supporting the involvement of ferroptosis in IDD and highlighting key DE-FRGs as potential therapeutic targets.

### 3.6 Immune microenvironment characteristics in IDD

**FIGURE 8:**
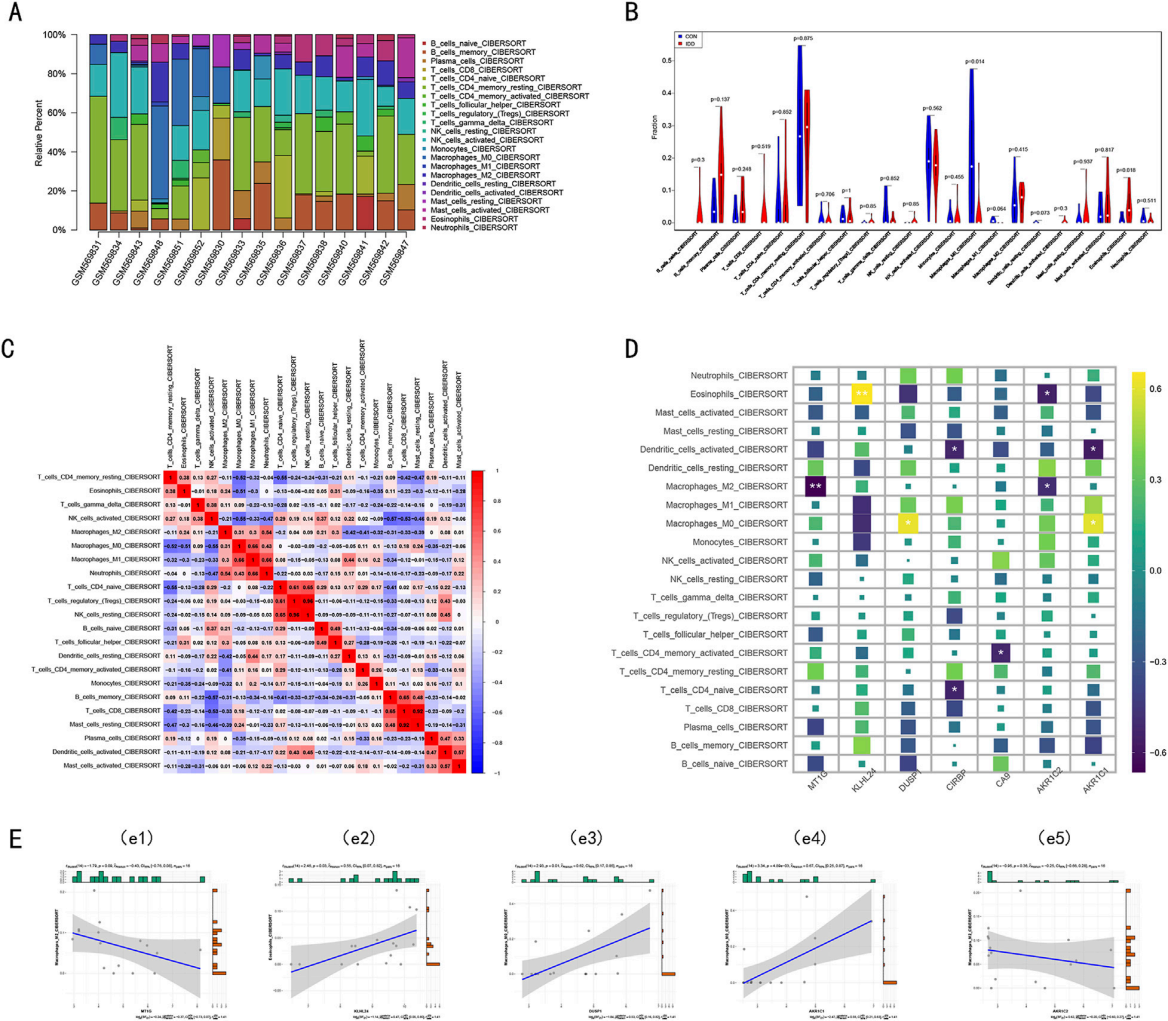
The immune infiltration landscape in the Intervertebral disc. **(A)** The stacked bar chart shows the distribution of 22 immune cells in the GSE23130 dataset, each bar represents one sample. **(B)** The violin plot demonstrates the difference in immune infiltration between intervertebral disc degenerative tissue and control tissue. **(C)** Correlation heat maps display the correlation matrix of 22 immune cell type proportions. Red: Positive correlation; Blue: Negative correlation. **(D)** Heatmap showed the correlations between the seven key DE-FRGs and 22 types of immune cells. Yellow: Upregulation; Purple: Downregulation. The depth of the colors represented the strength of the correlation. **(E)** Correlation analysis between immune cell infiltration and the expression of key DE-FRGs.

The etiology of IDD is thought to involve immune dysregulation within the disc microenvironment. To explore the immune system’s potential role in IDD, we analyzed immune cell infiltration in IDD samples using the GSE23130 dataset and the CIBERSORT algorithm. Our findings revealed significant differences in immune cell infiltration between IDD and control (CON) groups, underscoring the possible contribution of immune factors to the development of IDD. Specifically, there was a marked increase in the infiltration of M0 macrophages and eosinophils in the IDD group (Figures 8A, B), indicating an altered immune landscape. Further analysis using a correlation matrix of the 22 immune cell subtypes revealed a positive correlation between M0 macrophages and other inflammatory cells such as M1 and M2 macrophages, as well as neutrophils. This suggests a complex network of immune interactions contributing to inflammation and possibly disc degeneration. Conversely, there was an inverse relationship observed between resting memory CD4+ T cells, activated NK cells, and eosinophils, highlighting the diverse roles of immune cells in IDD. Interestingly, eosinophils, which were elevated in IDD, also showed positive correlations with resting memory CD4+ T cells, M2 macrophages, and T follicular helper cells (Figure 8C), further illustrating their potential role in modulating immune responses in IDD. Acknowledging the pivotal role of diverse immune components in diagnosing IDD and its underlying pathogenic mechanisms, we delved into the association between immune cell infiltration and the expression of key DE-FRGs. Notably, *DUSP1* and *AKR1C1* were found to be upregulated in M0 macrophages. In eosinophils, *KLHL24* expression was markedly elevated (*P* < 0.01), whereas *AKR1C2* displayed a declining trend (*P* < 0.05) (Figures 8D, E).

These findings suggest that specific immune cells, particularly M0 macrophages and eosinophils, may influence the expression of genes related to ferroptosis, potentially linking the immune response to iron-dependent cell death in IDD. This connection could be key in understanding the pathogenic mechanisms of IDD and could lead to targeted therapies that address these immune and molecular interactions.

## 4 Discussion

IDD is predominantly considered a degenerative disease and is the primary cause of chronic lower back pain (Takatalo et al., 2012; Manchikanti et al., 2009). Various pathogenic factors can disrupt the standard activity and function of both nucleus pulposus (NP) cells and annulus fibrosus (AF) cells, culminating in the deterioration of the intervertebral disc (Lotz and Ulrich, 2006; Urban, 2002). Serving as the central structural and functional component of the intervertebral disc (IVD), the NP often undergoes pathological changes early in IDD. Research has shown that cell mortality rates in degenerative human intervertebral disc samples fluctuate between 53% and 73%, particularly within the NP region. Surrounding the NP, the annulus fibrosus connects adjacent vertebral bones and faces degeneration primarily due to the loss of fibrous ring cells and imbalances in collagen fiber synthesis and metabolism (Fontana et al., 2015; Sakai and Grad, 2015; Colombini et al., 2008). Recent research has emphasized preserving IVD cell activity, with increasing interest in the potential relationship between ferroptosis and IDD (Dou et al., 2023; Ke et al., 2023; Li et al., 2023).

In this study, we explored the role of ferroptosis in the pathophysiology of IDD, specifically investigating its impact on NP and AF cells. Through bioinformatics analysis, we examined the GSE23130 dataset, pinpointing 68 potential ferroptosis-related genes (FRGs) associated with IDD. We subsequently subjected these differentially expressed FRGs to enrichment analysis to determine their possible biological roles. Our Gene Ontology (GO) examination of the FRGs identified various enrichment categories linked to ferroptosis, including macroautophagy, mitophagy, collagen protein metabolic processes, and the regulation of fibroblast proliferation. The GSEA demonstrate an increased activation of pathways involved in ferroptosis, ROS production, and immune infiltration, aligning with recent studies that underscore the multifactorial nature of IDD. This correlation supports the hypothesis that oxidative stress and immune responses play a crucial role in disc degeneration. Several signaling channels recognized through the Kyoto Encyclopedia of Genes and Genomes (KEGG) examination, like PI3K-AKT, P38 MAPK, TGF-β, and mTOR, correlate with ferroptosis. To differentiate between FRGs in the NP and the AF, we analyzed the GSE70362 dataset, cross-referencing our findings with the identified FRGs. This analysis unveiled 45 FRGs in the NP and 26 in the AF. When comparing both the GSE23130 and GSE70362 datasets, we identified seven DE-FRGs. Notably, *MT1G* and *CA9* appeared in both NP and AF datasets, *AKR1C1*, *AKR1C2*, *DUSP1*, and *CIRBP* were specific to NP, and *KLHL24* was exclusive to AF. The consistent expression of *MT1G* and *CA9* in both NP and AF tissues across different stages of IDD suggests these proteins could serve as viable biomarkers for early diagnosis and progression monitoring of the disease. Future clinical trials could explore the efficacy of targeting these markers to slow or reverse disc degeneration.

The validation of our findings through the single-cell database (GSE199866) is crucial for confirming the specificity of gene expression changes in IDD. This analysis enables us to identify the precise cell types showing ferroptosis-related alterations, adding a critical layer of validation to our bioinformatics findings. Additionally, we categorized classified clinical samples according to the lumbar intervertebral disc’s Pfirrmann grading. We also developed a mouse model for IDD, closely mimicking the pathological progression of human IDD induced by mechanical stress. The IVD samples from this IDD mouse model exhibited pronounced degeneration, reflecting the pathophysiology seen in human IDD. Notably, in both the advanced degenerative clinical samples and the IDD mouse specimens, we detected a marked decline in GPX4 protein expression. This indicates a potential surge in ferroptosis as IDD progresses (Chen X. et al., 2021). Building upon our bioinformatics findings, we utilized immunohistochemistry (IHC) to validate the expression of the seven identified differentially expressed FRGs in both clinical samples and the IDD mouse model. Experimental results confirmed that *MT1G*, *CA9*, *KLHL24*, AKRC1R1, AKRC1R2, and *DUSP1* protein expression patterns aligned with our bioinformatics insights.

*MT1G* is a member of the Metallothionein (MTs) family (Mehus et al., 2014). This gene encodes a compact, cysteine-rich protein that play an important role in protecting cells from heavy metal toxicity, oxidative stress damage, and are involved in cell proliferation and apoptosis. *MT1G*’s specific functions include regulating the availability of metal ions, acting as an antioxidant, and potentially participating in cell signaling pathways. Recent research suggests that its dysregulation is associated with various human malignancies and can suppress ferroptosis in tumors (Sun et al., 2016). In single-cell analyses focusing on IDD, elevated *MT1G* expression was identified in IDD tissues, suggesting that *MT1G* may influence IDD by mitigating calcification (Cherif et al., 2022; Li et al., 2022). In this study, we confirmed that *MT1G* is highly expressed during IDD, and the expression of *MT1G* is upregulated across various cell clusters in degenerated tissues, reflecting an attempt by cells to protect themselves and adapt to the degenerative environment. This widespread upregulation may indicate a link between disc degeneration and imbalances in metal ion homeostasis and increased oxidative stress. Given *MT1G*’s role in resisting oxidative stress and regulating metal ion balance, enhancing its expression or function could provide protection against disc degeneration. Potential therapeutic strategies might include promoting *MT1G* expression or adjusting metal ion balance to reduce oxidative damage and promote cell survival.

*CA9*, commonly known as Carbonic Anhydrase IX, is a transmembrane enzyme significantly induced under hypoxic conditions and plays a vital role in regulating the cellular acid-base balance (Li et al., 2020). Research has linked *CA9* with the process of ferroptosis, suggesting it could counteract this iron-dependent form of cell death in various cancers by modifying transferrin endocytosis and stabilizing ferritin levels (Zhang C. et al., 2023; Li et al., 2019). Furthermore, *CA9* is expressed in NP cells, with increased levels observed in conditions of heightened oxygen concentration, a state contrary to the cells’ naturally hypoxic environment (Silagi et al., 2018). As IDD progresses, these cells are increasingly exposed to lower pH levels and more oxygen, indicating *CA9*’s potential role in adjusting to these changes. The upregulation of *CA9* noted in our study, particularly in clusters associated with late-stage degeneration, supports its involvement in inflammatory responses, cell differentiation and growth, protein synthesis disruption, cell division, and tissue repair processes. This suggests that *CA9*’s expression might facilitate cellular adaptation to the hypoxic and acidic microenvironments during disc degeneration. Therefore, targeting *CA9* and strategies to improve the cellular micro-environment may offer new directions for the treatment of disc degeneration.

*AKR1C1* and *AKR1C2* are members of the aldo-keto reductase superfamily and serve as modulators of hormone activity. These enzymes catalyze the conversion of aldehydes and ketones into their respective alcohols, employing NADH and/or NADPH as cofactors (Detlefsen, 2023). Previous studies have shown that *AKR1C1* and *AKR1C2* can efficiently degrade lipid peroxides produced by 12/15-LOX, which enhances cellular resistance to drug-induced endoplasmic reticulum-induced ferroptosis (Gagliardi et al., 2019; Wohlhieter et al., 2020). Increased levels of *AKR1C1* and *AKR1C2* may play a protective role in IDD by effectively degrading lipid peroxides to counteract ferroptosis.

*DUSP1* is a dual-specific phosphatase that targets both tyrosine and serine residues (Bermudez et al., 2010). It acts on the MAP kinase MAPK1/ERK2, thereby playing a pivotal role in modulating the functions of the MAPK family, particularly in cancer cells. Recent studies suggest that *DUSP1* might curtail autophagosomes by inhibiting the phosphorylation of ULK1 or BECN1. This action establishes a feedback loop that dampens autophagy-dependent ferroptosis (Wang et al., 2016). Furthermore, investigations into the UBR3/*DUSP1* ubiquitination in IDD reveal that overexpression of UBR3 accelerates the ubiquitination and subsequent degradation of DSUP1 (Jiang et al., 2022). This degradation process results in suppressed cell growth, heightened cell apoptosis, and amplified inflammatory reactions in NP cells.

Cold-inducible RNA-binding protein (*CIRBP*) is recognized as a stress response protein that responds to a range of physiological and pathological stimuli, including hypoxia, ultraviolet radiation, glucose deprivation, heat stress, and disruptions in circadian rhythm (Zhong and Huang, 2017). In various tissues and under different stimuli, *CIRBP* plays a crucial role in attenuating cell apoptosis through pathways like p53, MAPK/ERK1/2, and the NF-κB pathway. Some research indicates that *CIRBP* can directly suppress the expression levels of p53, leading to the activation of the downstream ferroptosis pathway (Gao et al., 2022). Nonetheless, there is a lack of studies addressing *CIRBP*’s role in the framework of IDD.

*KLHL24*, an E3 ligase receptor responsible for substrate ubiquitination, can modulate its own ubiquitination (Cui et al., 2022). While it has been identified in tumor ferroptosis, its role in IDD remains intriguing, given the significant part ubiquitination plays during IDD progression. This positions *KLHL24* as a compelling subject for further investigation.

Due to its distinctive structure, the intervertebral disc is considered an immune-privileged organ. However, recent literature has underscored the critical influence of immune cell infiltration in IDD, with the connection between ferroptosis and this infiltration yet to be fully understood (Francisco et al., 2022). Utilizing the CiberSort method, we analyzed specific immune cell types in the intervertebral disc tissue derived from the GSE23130 dataset. Notably, the IDD group demonstrated markedly elevated M0 macrophages and eosinophils infiltration levels compared to the control group. This observation corroborates earlier findings, which posit that macrophages might amplify the immune cell recruitment to the intervertebral disc through cytokine release, thus hastening IDD progression.

Moreover, our correlation analysis between DE-FRGs and immune cells revealed increased expression of *DUSP1* and *AKR1C1* in M0 macrophages. *KLHL24* expression was heightened in eosinophils, whereas *AKR1C2* expression showed a downward trajectory within the same cell type. Past studies have suggested that *DUSP1* may modulate macrophage activation, operating as a principal downstream target protein of Ninj1, which subsequently can initiate inflammatory responses (Hu et al., 2023).

This study is accompanied by several limitations that warrant acknowledgment. Firstly, while we have ascertained the altered expression of key ferroptosis genes in IDD, we have not delved deeply into the underlying biological mechanisms linking ferroptosis to immune infiltration. Future clinical trials could explore the efficacy of targeting these markers to slow or reverse disc degeneration Secondly, the bipedal standing mouse model for lumbar disc degeneration, though designed to replicate human lumbar disc degeneration, is not without its discrepancies when juxtaposed with actual clinical specimens. Lastly, procuring normal human intervertebral disc tissues poses a formidable challenge. Consequently, we encountered difficulties obtaining clinical samples that fall within a Pfirrmann grade of 1. Such a limitation may circumscribe our research’s breadth and introduce biases or variations in our findings.

## Data Availability

The original contributions presented in the study are publicly available. This data can be found here: https://doi.org/10.6084/m9.figshare.26953588.v1.

## References

[B1] Andersson G. B. (1999). Epidemiological features of chronic low-back pain. Lancet 354 (9178), 581–585. 10.1016/S0140-6736(99)01312-4 10470716

[B2] Ao X. Wang L. Shao Y. Chen X. Zhang J. Chu J. (2019). Development and characterization of a novel bipedal standing mouse model of intervertebral disc and facet joint degeneration. Clin. Orthop. Relat. Res. 477 (6), 1492–1504. 10.1097/CORR.0000000000000712 31094848 PMC6554109

[B3] Bermudez O. Pagès G. Gimond C. (2010). The dual-specificity MAP kinase phosphatases: critical roles in development and cancer. Am. J. Physiol. Cell Physiol. 299 (2), C189–C202. 10.1152/ajpcell.00347.2009 20463170

[B4] Capossela S. Schläfli P. Bertolo A. Janner T. Stadler B. M. Pötzel T. (2014). Degenerated human intervertebral discs contain autoantibodies against extracellular matrix proteins. Eur. Cell Mater 27, 251–263. 10.22203/ecm.v027a18 24706108

[B5] Chen S. Chen M. Wu X. Lin S. Tao C. Cao H. (2021a). Global, regional and national burden of low back pain 1990-2019: a systematic analysis of the Global Burden of Disease study 2019. J. Orthop. Transl. 32, 49–58. 10.1016/j.jot.2021.07.005 PMC8639804 34934626

[B6] Chen X. Yu C. Kang R. Kroemer G. Tang D. (2021b). Cellular degradation systems in ferroptosis. Cell Death Differ. 28 (4), 1135–1148. 10.1038/s41418-020-00728-1 33462411 PMC8027807

[B7] Cherif H. Mannarino M. Pacis A. S. Ragoussis J. Rabau O. Ouellet J. A. (2022). Single-cell RNA-seq analysis of cells from degenerating and non-degenerating intervertebral discs from the same individual reveals new biomarkers for intervertebral disc degeneration. Int. J. Mol. Sci. 23 (7), 3993. 10.3390/ijms23073993 35409356 PMC8999935

[B8] Colombini A. Lombardi G. Corsi M. M. Banfi G. (2008). Pathophysiology of the human intervertebral disc. Int. J. Biochem. Cell Biol. 40 (5), 837–842. 10.1016/j.biocel.2007.12.011 18243770

[B9] Cui J. Zhao Q. Song Z. Chen Z. Zeng X. Wang C. (2022). *KLHL24*-Mediated hair follicle stem cells structural disruption causes alopecia. J. Invest Dermatol. 142 (8), 2079–2087.e8. 10.1016/j.jid.2022.01.007 35066002

[B10] Detlefsen A. J. (2023). Aldo keto-reductase family 1C members 1 through 4 recombinant enzyme purification and enzyme assay. Methods Enzymol. 689, 303–329. 10.1016/bs.mie.2023.04.007 37802576

[B11] Dou X. Ma Y. Luo Q. Song C. Liu M. Liu X. (2023). Therapeutic potential of melatonin in the intervertebral disc degeneration through inhibiting the ferroptosis of nucleus pulpous cells. J. Cell Mol. Med. 27 (16), 2340–2353. 10.1111/jcmm.17818 37329158 PMC10424295

[B12] Fontana G. See E. Pandit A. (2015). Current trends in biologics delivery to restore intervertebral disc anabolism. Adv. Drug Deliv. Rev. 84, 146–158. 10.1016/j.addr.2014.08.008 25174310

[B13] Francisco V. Pino J. González-Gay M. Á. Lago F. Karppinen J. Tervonen O. (2022). A new immunometabolic perspective of intervertebral disc degeneration. Nat. Rev. Rheumatol. 18 (1), 47–60. 10.1038/s41584-021-00713-z 34845360

[B14] Gagliardi M. Cotella D. Santoro C. Corà D. Barlev N. A. Piacentini M. (2019). Aldo-keto reductases protect metastatic melanoma from ER stress-independent ferroptosis. Cell Death Dis. 10 (12), 902. 10.1038/s41419-019-2143-7 31780644 PMC6883066

[B15] Gao H. Xie R. Huang R. Wang C. Wang Y. Wang D. (2022). *CIRBP* regulates pancreatic cancer cell ferroptosis and growth by directly binding to p53. J. Immunol. Res. 2022, 2527210. 10.1155/2022/2527210 36061308 PMC9436628

[B16] Ge H. Xue X. Xian J. Yuan L. Wang L. Zou Y. (2022). Ferrostatin-1 alleviates white matter injury via decreasing ferropto-sis following spinal cord injury. Mol. Neurobiol. 59, 161–176. 10.1007/s12035-021-02571-y 34635980

[B18] Hu Y. Zhan F. Wang Y. Wang D. Lu H. Wu C. (2023). The ninj1/*DUSP1* Axis contributes to liver ischemia reperfusion injury by regulating macro-phage activation and neutrophil infiltration. Cell Mol. Gastroenterol. Hepatol. 15 (5), 1071–1084. 10.1016/j.jcmgh.2023.01.008 36731792 PMC10036740

[B19] Jiang Z. Zhao Q. Chen L. Luo Y. Shen L. Cao Z. (2022). UBR3 promotes inflammation and apoptosis via *DUSP1*/p38 pathway in the nucleus pulposus cells of patients with intervertebral disc degeneration. Hum. Cell 35 (3), 792–802. 10.1007/s13577-022-00693-6 35332432

[B20] Kanehisa M. Goto S. (2000). KEGG: Kyoto Encyclopedia of genes and Genomes. Nucleic Acids Res. 28(1): 27–30. 10.1093/nar/28.1.27 10592173 PMC102409

[B21] Ke W. Liao Z. Liang H. Tong B. Song Y. Li G. (2023). Stiff substrate induces nucleus pulposus cell ferroptosis via YAP and N-cadherin mediated mechanotransduction. Adv. Healthc. Mater 12 (23), e2300458. 10.1002/adhm.202300458 37022980

[B22] Li S. Liao Z. Yin H. Liu O. Hua W. Wu X. (2023). G3BP1 coordinates lysophagy activity to protect against compression-induced cell ferroptosis during intervertebral disc degeneration. Cell Prolif. 56 (3), e13368. 10.1111/cpr.13368 36450665 PMC9977669

[B23] Li Z. Jiang L. Chew S. H. Hirayama T. Sekido Y. Toyokuni S. (2019). Carbonic anhydrase 9 confers resistance to ferroptosis/apoptosis in malignant mesothelioma under hypoxia. Redox Biol. 26, 101297. 10.1016/j.redox.2019.101297 31442913 PMC6831888

[B24] Li Z. Jiang L. Toyokuni S. (2020). Role of carbonic anhydrases in ferroptosis-resistance. Arch. Biochem. Biophys. 689, 108440. 10.1016/j.abb.2020.108440 32485154

[B25] Li Z. Ye D. Dai L. Xu Y. Wu H. Luo W. (2022). Corrigendum: single-cell RNA sequencing reveals the difference in human normal and degenerative nucleus pulposus tissue profiles and cellular interactions. Front. Cell Dev. Biol. 10, 1051707. 10.3389/fcell.2022.1051707 36313561 PMC9597866

[B26] Liberzon A. Subramanian A. Pinchback R. Thorvaldsdóttir H. Tamayo P. Mesirov J. P. (2011). Molecular signatures database (MSigDB) 3.0. Bioinformatics 27(12): 1739–1740. 10.1093/bioinformatics/btr260 21546393 PMC3106198

[B27] Lotz J. C. Ulrich J. A. (2006). Innervation, inflammation, and hypermobility may characterize pathologic disc degeneration: review of animal model data. J. Bone Jt. Surg. Am. 88 (Suppl. 2), 76–82. 10.2106/JBJS.E.01448 16595449

[B28] Ma X. Su J. Wang B. Jin X. (2022). Identification of characteristic genes in whole blood of intervertebral disc degeneration Pa-tients by weighted gene coexpression network analysis (WGCNA). Comput. Math. Methods Med. 2022, 6609901. 10.1155/2022/6609901 35069789 PMC8776439

[B29] Manchikanti L. Singh V. Datta S. Cohen S. P. Hirsch J. A. American Society of Interventional Pain Physicians (2009). Comprehensive review of epidemiology, scope and impact of spinal pain. Pain Physician 12, E35–E70. 19668291

[B30] Masaldan S. Bush A. I. Devos D. Rolland A. S. Moreau C. (2019). Striking while the iron is hot: iron metabolism and ferroptosis in neurodegeneration. Free Radic. Biol. Med. 133, 221–233. 10.1016/j.freeradbiomed.2018.09.033 30266679

[B31] Mehus A. A. Muhonen W. W. Garrett S. H. Somji S. Sens D. A. Shabb J. B. (2014). Quantitation of human metallothionein isoforms: a family of small, highly conserved, cysteine-rich proteins. Mol. Cell Proteomics 13 (4), 1020–1033. 10.1074/mcp.M113.033373 24493013 PMC3977181

[B32] Newman A. M. Liu C. L. Green M. R. Gentles A. J. Feng W. Xu Y. (2015). Robust enumeration of cell subsets from tissue expression profiles. Nat. Methods 12 (5), 453–457. 10.1038/nmeth.3337 25822800 PMC4739640

[B33] Pfirrmann C. W. Metzdorf A. Zanetti M. Hodler J. Boos N. (2001). Magnetic resonance classification of lumbar intervertebral disc degeneration. Spine (Phila Pa 1976) 26 (17), 1873–1878. 10.1097/00007632-200109010-00011 11568697

[B34] Ritchie M. E. Phipson B. Wu D. Hu Y. Law C. W. Shi W. (2015). Limma powers differential expression analyses for RNA-sequencing and microarray studies. Nucleic Acids Res. 43(7), e47, 10.1093/nar/gkv007 25605792 PMC4402510

[B35] Sakai D. Grad S. (2015). Advancing the cellular and molecular therapy for intervertebral disc disease. Adv. Drug Deliv. Rev. 84, 159–171. 10.1016/j.addr.2014.06.009 24993611

[B36] Silagi E. S. Schoepflin Z. R. Seifert E. L. Merceron C. Schipani E. Shapiro I. M. (2018). Bicarbonate recycling by HIF-1-Dependent carbonic anhydrase isoforms 9 and 12 is critical in maintaining intracellular pH and viability of nucleus pulposus cells. J. Bone Min. Res. 33 (2), 338–355. 10.1002/jbmr.3293 PMC5947995 28940640

[B37] Steenwyk J. L. Rokas A. (2021). Ggpubfigs: colorblind-friendly color palettes and ggplot2 graphic system extensions for publication-quality scientific figures. Microbiol. Resour. Announc. 10(44): e0087121-21. 10.1128/MRA.00871-21 34734767 PMC8567791

[B38] Subramanian A. Tamayo P. Mootha V. K. Mukherjee S. Ebert B. L. Gillette M. A. (2005). Gene set enrichment analysis: a knowledge-based approach for interpreting genome-wide expression profiles. Proc. Natl. Acad. Sci. 102(43): 15545–15550. 10.1073/pnas.0506580102 16199517 PMC1239896

[B39] Sun X. Niu X. Chen R. He W. Chen D. Kang R. (2016). Metallothionein-1G facilitates sorafenib resistance through inhibition of ferroptosis. Hepatology 64 (2), 488–500. 10.1002/hep.28574 27015352 PMC4956496

[B40] Takatalo J. Karppinen J. Niinimäki J. Taimela S. Mutanen P. Sequeiros R. B. (2012). Association of modic changes, Schmorl's nodes, spondylolytic defects, high-intensity zone lesions, disc herniations, and radial tears with low back symptom severity among young Finnish adults. Spine (Phila Pa 1976) 37 (14), 1231–1239. 10.1097/BRS.0b013e3182443855 22166927

[B41] Tam V. Chan W. C. W. Leung V. Y. L. Cheah K. S. E. Cheung K. M. C. Sakai D. (2018). Histological and reference system for the analysis of mouse intervertebral disc. J. Orthop. Res. 36 (1), 233–243. 10.1002/jor.23637 28636254

[B17] The Gene Ontology (GO) database and informatics resource[J]. Nucleic Acids Res., 2004, 32(90001): 258D - 261. 10.1093/nar/gkh036 PMC308770 14681407

[B42] Urban J. P. (2002). The role of the physicochemical environment in determining disc cell behaviour. Biochem. Soc. Trans. 30 (Pt 6), 858–864. 10.1042/bst0300858 12440933

[B43] Wang J. Zhou J. Y. Kho D. Reiners J. J. Jr Wu G. S. (2016). Role for *DUSP1* (dual-specificity protein phosphatase 1) in the regulation of autophagy. Autophagy 12 (10), 1791–1803. 10.1080/15548627.2016.1203483 27459239 PMC5079544

[B44] Wang L. He T. Liu J. Tai J. Wang B. Zhang L. (2021). Revealing the immune infiltration landscape and identifying diagnostic biomarkers for lumbar disc herniation. Front. Immunol. 12, 666355. 10.3389/fimmu.2021.666355 34122424 PMC8190407

[B45] Wang W. Jing X. Du T. Ren J. Liu X. Chen F. (2022). Iron overload promotes intervertebral disc degeneration via inducing oxidative stress and ferroptosis in endplate chondrocytes. Free Radic. Biol. Med. 190, 234–246. 10.1016/j.freeradbiomed.2022.08.018 35981695

[B46] Wei T. Zhang M. Zheng X. Xie T. Wang W. Zou J. (2022). Interferon‐γ induces retinal pigment epithelial cell Ferroptosis by a JAK1‐2/STAT1/SLC7A11 signaling pathway in Age‐related Macular Degeneration. FEBS J. 289, 1968–1983. 10.1111/febs.16272 34741776

[B47] Weiler C. Lopez-Ramos M. Mayer H. M. Korge A. Siepe C. J. Wuertz K. (2011). Histological analysis of surgical lumbar intervertebral disc tissue provides evidence for an association between disc degeneration and increased body mass index. BMC Res. Notes 4, 497. 10.1186/1756-0500-4-497 22087871 PMC3226673

[B48] Wohlhieter C. A. Richards A. L. Uddin F. Hulton C. H. Quintanal-Villalonga À. Martin A. (2020). Concurrent mutations in STK11 and KEAP1 promote ferroptosis protection and SCD1 dependence in lung cancer. Cell Rep. 33 (9), 108444. 10.1016/j.celrep.2020.108444 33264619 PMC7722473

[B49] Xin J. Wang Y. Zheng Z. Wang S. Na S. Zhang S. (2022). Treatment of intervertebral disc degeneration. Orthop. Surg. 14, 1271–1280. 10.1111/os.13254 35486489 PMC9251272

[B50] Yang R. Xu W. Zheng H. Zheng X. Li B. Jiang L. (2021). Involvement of oxidative stress‐induced annulus fibrosus cell and nucleus pulposus cell ferroptosis in intervertebral disc degeneration pathogenesis. J. Cell. Physiology 236, 2725–2739. 10.1002/jcp.30039 PMC7891651 32892384

[B51] Ye F. Lyu F. J. Wang H. Zheng Z. (2022). The involvement of immune system in intervertebral disc herniation and degeneration. JOR Spine 5 (1), e1196. 10.1002/jsp2.1196 35386754 PMC8966871

[B52] Yu G. Wang L. G. Han Y. He Q. Y. (2012). clusterProfiler: an R Package for comparing biological themes among gene clusters. OMICS A J. Integr. Biol. 16 (5): 284–287. 10.1089/omi.2011.0118 PMC3339379 22455463

[B53] Yu X. Xu H. Liu Q. Wang Y. Wang S. Lu R. (2022). circ_0072464 shuttled by bone mesenchymal stem cell-secreted extracel-lular vesicles inhibits nucleus pulposus cell ferroptosis to relieve intervertebral disc degeneration. Oxidative Med. Cell. Longev. 2022, 1–21. 10.1155/2022/2948090 PMC9259290 35814268

[B54] Zhang C. Lu X. Liu X. Xu J. Li J. Qu T. (2023b). Carbonic anhydrase IX controls vulnerability to ferroptosis in gefitinib-resistant lung cancer. Oxid. Med. Cell Longev. 2023, 1367938. 10.1155/2023/1367938 36760347 PMC9904911

[B55] Zhang F. Cui D. Wang K. Cheng H. Zhai Y. Jiao W. (2023a). Identifification and validation of ferroptosis signatures and immune infifiltration characteristics associated with intervertebral disc degeneration. Front. Genet. 14, 1133615. 10.3389/fgene.2023.1133615 36911415 PMC9992550

[B56] Zhang Y. Han S. Kong M. Tu Q. Zhang L. Ma X. (2021). Single-cell RNA-seq analysis identifies unique chondrocyte subsets and reveals involvement of ferroptosis in human intervertebral disc degeneration. Osteoarthr. Cartil. 29 (9), 1324–1334. 10.1016/j.joca.2021.06.010 34242803

[B57] Zhong P. Huang H. (2017). Recent progress in the research of cold-inducible RNA-binding protein [published correction appears in Future Sci OA. 2018 Apr 11;4(5):FSO246]. Future Sci. OA 3 (4), FSO246. 10.4155/fsoa-2017-0077 29134130 PMC5674272

