# Supplementary material for: Identification and validation of ferroptosis-related biomarkers in intervertebral disc degeneration
Source: Front Cell Dev Biol. 2024 Sep 16;12:1416345. doi: 10.3389/fcell.2024.1416345 (PMC11439793; doi:10.3389/fcell.2024.1416345)
Supplement: Supplementary file 1 [file Table1.DOCX]

**SUPPLEMENTARY TABLE 1** The 68 Differentially Expressed FRGs in the microarray data GSE23130

| **Gene Symbol** | **LogFC** | **Changes** | ***P*-value** |
| --- | --- | --- | --- |
| *FTL*  *H19*  *NNMT*  *CFL1*  *TIMP1*  *GABARAPL2*  *RPL8*  *CAV1*  *SQSTM1*  *GJA1*  *AKR1C2*  *IDH1*  *SAT1*  *TMSB4X*  *UBC*  *XBP1*  *HIF1A*  *EGR1*  *PARK7*  *HSPB1*  *DUSP1*  *AKR1C1*  *NCOA4*  *BNIP3*  *VDAC2*  *TSC22D3*  *CIRBP*  *MT1G*  *PRDX6*  *TXN*  *SLC39A14*  *TMBIM4*  *PRDX1*  *IDH2*  *STEAP3*  *MTDH*  *ZFP36*  *P4HB*  *EPAS1*  *CS*  *CA9*  *YWHAE*  *HSPA5*  *PTEN*  *MFN2*  *TXNIP*  *COPZ1*  *QSOX1*  *SLC38A1*  *VCP*  *MAPK14*  *KLHDC3*  *YAP1*  *CHMP1A*  *USP7*  *MMP13*  *PSAT1*  *ELOVL5*  *CHMP5*  *CP*  *MYB*  *PTPN18*  *TFR2*  *KLHL24*  *WIPI2*  *KRAS*  *PPARD*  *LPCAT3* | 5.388117927  4.81272252  4.006830521  3.719999053  3.332447125  3.1257415  2.967265729  2.688283792  2.680214708  2.648112958  2.452671406  2.443261063  2.410816021  2.353768042  2.312528167  2.302765469  2.177184115  2.1375025  2.120814563  2.12031176  2.087124781  2.079943948  2.062238677  2.007647604  1.971932948  1.955561083  1.947453542  1.839477854  1.742645927  1.727915198  1.704431813  1.703214302  1.661007646  1.64755426  1.626327187  1.448799802  1.439272167  1.373505073  1.357764323  1.33012499  1.324617771  1.288083427  1.278225573  1.2781975  1.265028438  1.239679719  1.225639729  1.216213875  1.212129646  1.149499969  1.129423677  1.128036198  1.115745604  1.106120146  1.054865229  1.036841396  1.017140438  1.001979198  1.001762063  -1.071791302  -1.107744917  -1.628626542  -1.836756521  -2.032709668  -2.175871979  -3.057261021  -3.461501001  -3.565318501 | up  up  up  up  up  up  up  up  up  up  up  up  up  up  up  up  up  up  up  up  up  up  up  up  up  up  up  up  up  up  up  up  up  up  up  up  up  up  up  up  up  up  up  up  up  up  up  up  up  up  up  up  up  up  up  up  up  up  up  down  down  down  down  down  down  down  down  down | 1.06E-05  0.019390729  0.001028746  0.008470741  0.001078658  4.80E-05  0.000365253  0.001049517  0.000156138  9.34E-05  0.000329818  0.021512583  0.001536575  0.013403486  0.001874401  0.022848694  0.000100551  0.024609793  0.010569965  0.001018641  1.44E-05  4.23E-05  1.52E-06  0.000707996  1.99E-05  0.004376082  0.007827976  0.004158935  0.00295286  0.008586007  0.005446964  0.000114436  0.005867522  0.013603852  2.28E-07  0.001225788  3.48E-05  0.007992254  0.001015552  0.001485082  0.033194343  0.007668867  0.010820601  0.000389173  0.001828154  0.000369601  0.000316342  0.000163072  0.00042879  1.20E-05  0.037277193  0.039797231  0.000504584  0.00095259  0.00226246  0.04926335  0.001125484  5.26E-05  0.000746307  0.00023852  0.019476546  1.07E-06  0.005107579  9.49E-06  0.00015215  0.000381478  0.003773469  0.000672511 |
