# Supplementary material for: Identification and validation of ferroptosis-related biomarkers in intervertebral disc degeneration
Source: Front Cell Dev Biol. 2024 Sep 16;12:1416345. doi: 10.3389/fcell.2024.1416345 (PMC11439793; doi:10.3389/fcell.2024.1416345)
Supplement: Supplementary file 2 [file Table8.docx]

**SUPPLEMENTARY TABLE 8** Data between the NC and IDD groups.

| **Variable mild-IDD（n=10） sereve-IDD（n=10） P-value** |
| --- |
| **Age（years） 38.1±10.43 65.9±14.65 p＜0.001**  **Gender（male：female） 3：7 3：7 -**  **Pfirrmann score（1-5） 2.7±0.13 4.8±0.16 p＜0.001** |
