# Supplementary figures and images for: Identification and validation of ferroptosis-related biomarkers in intervertebral disc degeneration
Source: Front Cell Dev Biol. 2024 Sep 16;12:1416345. doi: 10.3389/fcell.2024.1416345 (PMC11439793; doi:10.3389/fcell.2024.1416345)

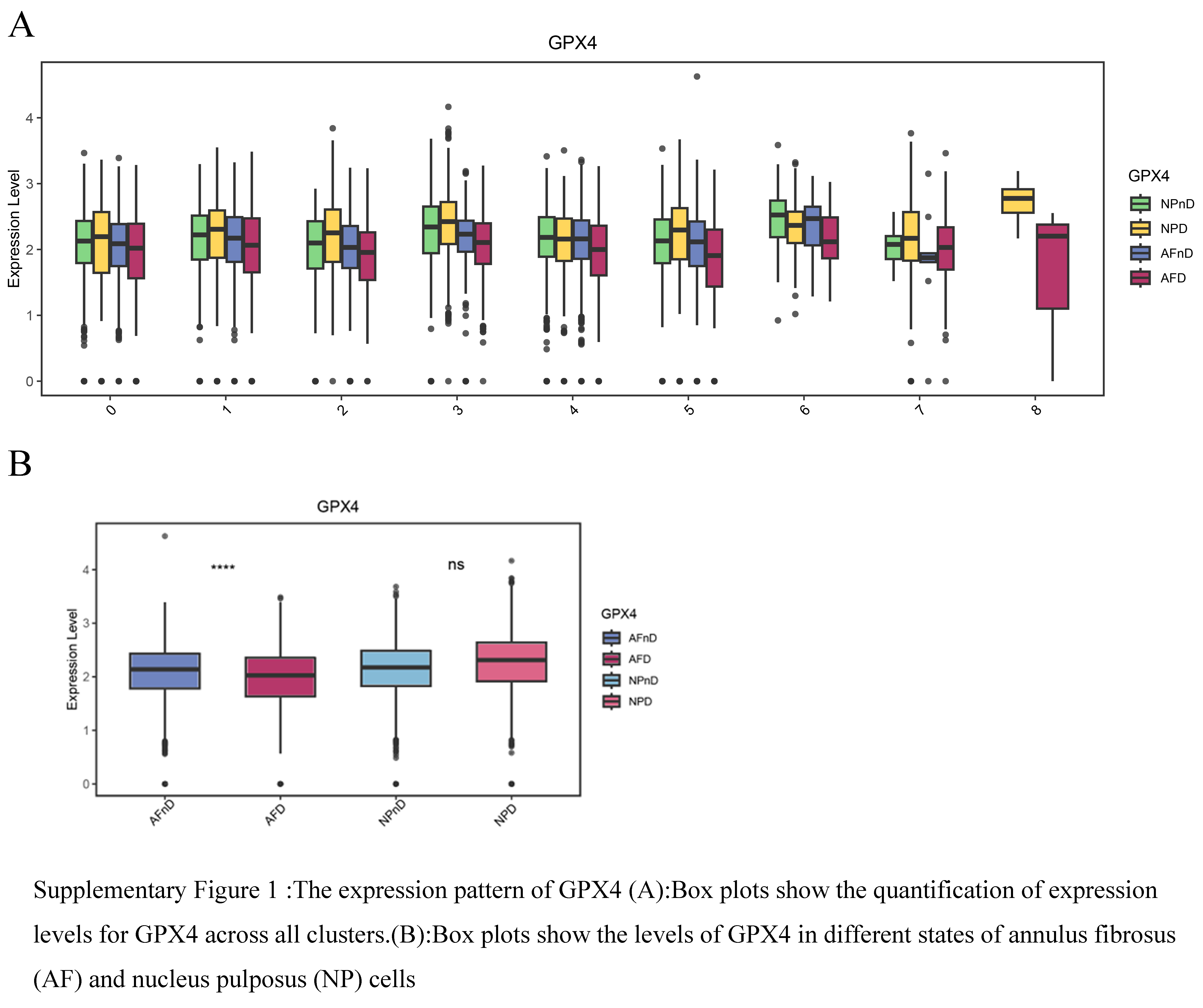

Supplement: Supplementary file 4 [file Image1.tif]
