# Supplementary material for: Identification and validation of ferroptosis-related biomarkers in intervertebral disc degeneration
Source: Front Cell Dev Biol. 2024 Sep 16;12:1416345. doi: 10.3389/fcell.2024.1416345 (PMC11439793; doi:10.3389/fcell.2024.1416345)
Supplement: Supplementary file 5 [file Table2.DOCX]

**TABLE 2 The Results of KEGG Enrichment Analysis of DEGs related to Ferroptosis.**

KEGG, Kyoto Encyclopedia of Genes and Genomes; IDD, ; BP, biological process; CC, cellular component; MF, molecular function.

| **Term** | **Description** | **Gene Ratio** | ***P-*value** |
| --- | --- | --- | --- |
| hsa04216 | Ferroptosis | 8/57 | 2.71228E-10 |
| hsa04621 | NOD-like receptor signaling pathway | 7/57 | 0.000268899 |
| hsa04068 | FoxO signaling pathway | 6/57 | 0.000282404 |
| hsa01210 | 2-Oxocarboxylic acid metabolism | 3/57 | 0.000288863 |
| hsa04218 | Cellular senescence | 5/57 | 0.004495524 |
| hsa04217 | Necroptosis | 5/57 | 0.004873152 |
| hsa04370 | VEGF signaling pathway | 3/57 | 0.007956084 |
| hsa04137 | Mitophagy - animal | 7/57 | 5.7383E-07 |
