# Supplementary material for: Identification and validation of ferroptosis-related biomarkers in intervertebral disc degeneration
Source: Front Cell Dev Biol. 2024 Sep 16;12:1416345. doi: 10.3389/fcell.2024.1416345 (PMC11439793; doi:10.3389/fcell.2024.1416345)
Supplement: Supplementary file 6 [file Table5.DOCX]

**SUPPLEMENTARY TABLE 5** The 26 Differentially Expressed FRGs in AF

| **Gene Symbol** | **LogFC** | **Changes** | ***P*-value** |
| --- | --- | --- | --- |
| MGST1  GDF15  TF  NQO1  TRIB3  MMD  PGD  HSD17B11  LIFR  PIR  DPP4  PLIN2  WIPI1  DNAJB6  PDK4  BID  KLHL24  GSTZ1  DDR2  KLF2  MUC1  ALOX12  CREB5  BEX1  MT1G  CA9 | 2.489137789  2.097114411  1.811713574  1.520245447  1.265613186  1.18398121  1.132030878  0.974576803  0.936448606  0.934393789  0.922823492  0.884751721  0.760657685  0.696649207  0.652206814  0.599976797  0.597030439  -0.612345071  -0.676701589  -0.705832845  -0.765373427  -0.83975771  -1.089715282  -1.174938236  -1.326276527  -1.54712472 | up  up  up  up  up  up  up  up  up  up  up  up  up  up  up  up  up  down  down  down  down  down  down  down  down  down | 7.98E-07  0.001245027  0.019983443  0.006045592  0.000754604  0.000211337  0.002241707  0.029874718  0.000838583  0.0470963  0.019850559  0.007369305  0.003312049  0.008807109  0.023012238  0.000302091  0.003219179  0.009702414  0.040542177  0.011463044  0.00098226  0.006516542  0.007771732  0.000513214  0.00508215  0.004639919 |
