# Supplementary material for: Identification and validation of ferroptosis-related biomarkers in intervertebral disc degeneration
Source: Front Cell Dev Biol. 2024 Sep 16;12:1416345. doi: 10.3389/fcell.2024.1416345 (PMC11439793; doi:10.3389/fcell.2024.1416345)
Supplement: Supplementary file 7 [file Table3.DOCX]

**TABLE 3 The Results of GO Enrichment Analysis of DEGs related to Ferroptosis.**

GO, Gene ontology; IDD, ; BP, biological process; CC, cellular component; MF, molecular function.

| **Ontology** | **Term** | **Description** | **Gene Ratio** | ***P-*value** |
| --- | --- | --- | --- | --- |
| BP | GO:0006879 | cellular iron ion homeostasis | 6/68 | 1.36953E-07 |
| BP | GO:0046916 | cellular transition metal ion homeostasis | 7/68 | 1.89904E-07 |
| BP | GO:0070233 | negative regulation of T cell apoptotic process | 2/68 | 0.002348363 |
| BP | GO:0050727 | regulation of inflammatory response | 6/68 | 0.002989042 |
| BP | GO:0019217 | regulation of fatty acid metabolic process | 3/68 | 0.004517154 |
| BP | GO:1902686 | mitochondrial outer membrane permeabilization involved in programmed cell death | 2/68 | 0.010579511 |
| BP | GO:0061001 | regulation of dendritic spine morphogenesis | 2/68 | 0.011057628 |
| BP | GO:0006749 | glutathione metabolic process | 2/68 | 0.023160566 |
| CC | GO:0005741 | mitochondrial outer membrane | 5/67 | 0.000681271 |
| CC | GO:0060205 | cytoplasmic vesicle lumen | 8/67 | 1.46443E-05 |
| CC | GO:0031983 | vesicle lumen | 8/67 | 1.53066E-05 |
| CC | GO:1904813 | ficolin-1-rich granule lumen | 3/67 | 0.008827619 |
| CC | GO:0031300 | intrinsic component of organelle membrane | 5/67 | 0.013344919 |
| CC | GO:0005770 | late endosome | 5/67 | 0.002823295 |
| MF | GO:0016209 | antioxidant activity | 4/66 | 0.000245258 |
| MF | GO:0051059 | NF-kappaB binding | 2/66 | 0.005504315 |
| MF | GO:0033764 | steroid dehydrogenase activity, acting on the CH-OH group of donors, NAD or NADP as acceptor | 2/66 | 0.005161076 |
| MF | GO:0001221 | transcription coregulator binding | 3/66 | 0.006872463 |
| MF | GO:0097110 | scaffold protein binding | 3/66 | 0.001789709 |
| MF | GO:1990381 | ubiquitin-specific protease binding | 3/66 | 5.58892E-05 |
