# Supplementary material for: Identification and validation of ferroptosis-related biomarkers in intervertebral disc degeneration
Source: Front Cell Dev Biol. 2024 Sep 16;12:1416345. doi: 10.3389/fcell.2024.1416345 (PMC11439793; doi:10.3389/fcell.2024.1416345)
Supplement: Supplementary file 8 [file Table4.DOCX]

**TABLE 4 The Results of GSEA of DEGs in IDD.**

GSEA, Gene set enrichment analysis; NES, normalized enrichment score; FDR, false discovery rate.

| **ID** | **NES** | **FDR** | ***P-*value** |
| --- | --- | --- | --- |
| GOBP_RESPONSE_TO_OXIDATIVE_STRESS | 1.41138689 | 0.010560108 | 0.000314983 |
| GOBP_REACTIVE_OXYGEN_SPECIES_METABOLIC_PROCESS | 1.545834319 | 0.008174778 | 0.000226953 |
| GOMF_ANTIOXIDANT_ACTIVITY | 1.607978958 | 0.039003071 | 0.001889632 |
| GOBP_SKELETAL_SYSTEM_DEVELOPMENT | 1.652312 | 4.04376E-08 | 1.46412E-10 |
| GOBP_BONE_DEVELOPMENT | 1.790742468 | 3.31547E-06 | 2.51518E-08 |
| GOBP_OSSIFICATION | 1.639404596 | 5.88466E-06 | 4.87006E-08 |
| GOCC_COLLAGEN_CONTAINING_EXTRACELLULAR_MATRIX | 2.106146126 | 0.000000029 | 1E-10 |
| GOMF_EXTRACELLULAR_MATRIX_STRUCTURAL_CONSTITUENT_CONFERRING_COMPRESSION_RESISTANCE | 2.030310401 | 7.63083E-07 | 4.21011E-09 |
| GOMF_EXTRACELLULAR_MATRIX_STRUCTURAL_CONSTITUENT | 2.188000354 | 0.000000029 | 1E-10 |
| GOMF_COLLAGEN_BINDING | 2.105206767 | 9.15585E-08 | 4.10434E-10 |
| GOBP_IRON_ION_HOMEOSTASIS | 1.493181907 | 0.116156994 | 0.010273886 |
| GOBP_RESPONSE_TO_METAL_ION | 1.529161601 | 0.000979806 | 1.74E-05 |
| GOBP_GLUTATHIONE_METABOLIC_PROCESS | 1.721968994 | 0.013139559 | 0.000446292 |
| GOBP_GLUTAMATE_RECEPTOR_SIGNALING_PATHWAY | -1.762393212 | 0.033028518 | 0.001464351 |
| GOBP_T_CELL_CYTOKINE_PRODUCTION | 1.700725771 | 0.04805338 | 0.002543515 |
| GOBP_INNATE_IMMUNE_RESPONSE_IN_MUCOSA | 1.758490349 | 0.008010275 | 0.000220973 |
| GOBP_HUMORAL_IMMUNE_RESPONSE | 1.406169302 | 0.065667764 | 0.004020505 |
| GOBP_HUMORAL_IMMUNE_RESPONSE_MEDIATED_BY_CIRCULATING_IMMUNOGLOBULIN | 1.515576292 | 0.108783646 | 0.009059052 |
