# Supplementary material for: Identification and validation of ferroptosis-related biomarkers in intervertebral disc degeneration
Source: Front Cell Dev Biol. 2024 Sep 16;12:1416345. doi: 10.3389/fcell.2024.1416345 (PMC11439793; doi:10.3389/fcell.2024.1416345)
Supplement: Supplementary file 9 [file Table6.DOCX]

**SUPPLEMENTARY TABLE 6** The 45 Differentially Expressed FRGs in NP

| **Gene Symbol** | **LogFC** | **Changes** | ***P*-value** |
| --- | --- | --- | --- |
| AKR1C3  NQO1  MGST1  SLC40A1  AKR1C1  ALDH3A2  MAP3K5  GDF15  PGD  BID  SLC2A12  MMD  LIFR  PIR  DNAJB6  AIFM2  GPX4  AKR1C2  TRIB2  SRXN1  NEDD4  PDK4  ABCC1  NOX4  ACO1  ISCU  PLIN2  MTOR  FURIN  DDIT3  CIRBP  BCAT2  ACSL1  AHCY  CAPG  DUSP1  GSTZ1  ATF3  JUN  MUC1  PROK2  BEX1  CA9  ENPP2  MT1G | 3.028269421  2.286389292  1.603302577  1.530507562  1.519217465  1.498924458  1.390523263  1.369902934  1.305864619  1.08007985  1.063307303  1.036409626  0.902881245  0.880267647  0.852314079  0.844274622  0.79637214  0.782850037  0.772487029  0.746073526  0.735132894  0.733183038  0.720535684  0.682052213  0.669545248  0.667246963  0.663213754  0.616994804  0.606685383  -0.586517192  -0.60047518  -0.63031093  -0.641344966  -0.651522081  -0.653165268  -0.672761273  -0.706022598  -0.725974647  -0.775424083  -0.80263087  -1.004682285  -1.312087293  -1.386209166  -1.582218269  -2.048914686 | up  up  up  up  up  up  up  up  up  up  up  up  up  up  up  up  up  up  up  up  up  up  up  up  up  up  up  up  up  down  down  down  down  down  down  down  down  down  down  down  down  down  down  down  down | 2.44E-06  0.000519792  1.57E-06  2.17E-05  2.11E-05  1.08E-05  0.001588299  0.01555408  0.000976367  0.000241466  0.005656166  0.00083621  0.01016525  0.003175125  0.024416582  0.000150195  8.61E-05  0.00108212  0.036394928  0.043120616  0.012366126  0.016274462  0.001193352  0.021838457  0.000377556  4.58E-05  0.033296378  0.024930955  0.035284812  0.015998821  0.020129569  0.001292603  0.017955915  0.016537371  0.032486404  0.04234253  0.001348525  0.010912462  0.017057606  9.43E-06  2.00E-05  0.000153557  0.001065271  6.20E-05  0.000213694 |
